# Tissue adhesive indocyanine green‐locking granular gel‐mediated photothermal therapy combined with checkpoint inhibitor for preventing postsurgical recurrence and metastasis of colorectal cancer

**DOI:** 10.1002/btm2.10576

**Published:** 2023-09-18

**Authors:** Yuan Zeting, Ma Shuli, Li Yue, Fang Haowei, Shang Jing, Zhan Yueping, Wang Jie, Chen Teng, Deng Wanli, Kunxi Zhang, Yin Peihao

**Affiliations:** ^1^ Interventional Cancer Institute of Chinese Integrative Medicine & Putuo Hospital Shanghai University of Traditional Chinese Medicine Shanghai P. R. China; ^2^ Central Laboratory, Putuo Hospital Shanghai University of Traditional Chinese Medicine Shanghai P. R. China; ^3^ Department of Pharmaceutics, School of Pharmacy East China University of Science and Technology Shanghai China; ^4^ Shanghai Putuo Central School of Clinical Medicine Anhui Medical University Hefei P. R. China; ^5^ Department of Polymer Materials, School of Materials Science and Engineering Shanghai University Shanghai P. R. China; ^6^ Department of General Surgery, Putuo Hospital Shanghai University of Traditional Chinese Medicine Shanghai P. R. China; ^7^ Department of Oncology, Putuo Hospital Shanghai University of Traditional Chinese Medicine Shanghai P. R. China

**Keywords:** colorectal cancer, granular hydrogel, ICG, immunotherapy, photothermal therapy

## Abstract

Developing effective therapy to inhibit postoperative recurrence and metastasis of colorectal cancer (CRC) is challenging and significant to reduce mortality and morbidity. Here, a granular hydrogel, assembled from gelatin microgels by dialdehyde starch and interpenetrated with in situ polymerized poly(sulfobetaine methacrylate‐*co*‐N‐isopropylacrylamide) (P(SBMA‐*co*‐NIPAM)), is prepared to load and lock Food and Drug Administration (FDA)‐approved indocyanine green (ICG) with definite photothermal function and biosafety for photothermal therapy (PTT) combining with checkpoint inhibitor. The presence of P(SBMA‐*co*‐NIPAM) endows granular hydrogel with high retention to water‐soluble ICG, preventing easy diffusion and rapid scavenging of ICG. The ICG‐locking granular hydrogel can be spread and adhered onto the surgery site at wet state in vivo, exerting a persistent and stable PTT effect. Combined with αPD‐L1 treatment, ICG‐locking granular hydrogel‐mediated PTT can eradicate postsurgery residual and metastatic tumors, and prevent long‐term tumor recurrence. Further mechanistic studies indicate that combination treatment effectively promotes dendritic cells maturation in lymph nodes, enhances the number and infiltration of CD8^+^ T and CD4^+^ T cells in tumor tissue, and improves memory T cell number in spleen, thus activating the antitumor immune response. Overall, ICG‐locking gel‐mediated PTT is expected to exhibit broad clinical applications in postoperative treatment of cancers, like CRC.

**Translational Impact Statement**

This work demonstrates the application of ICG, the clinical photothermal agent, in postoperative treatment of colorectal cancer. The designed granular hydrogel not only prevents the diffusion of hydrophilic ICG to guarantee the PTT effect but also adheres stably to the whole wound area at body temperature. Therefore, ICG‐locking gel‐mediated PTT combined with a PD‐L1 treatment is expected to exhibit broad clinical applications in the postoperative treatment of cancers like CRC.

## 1 INTRODUCTION

Surgery resection is the first‐line treatment for colorectal cancer (CRC), which is the most common malignant tumor of the digestive system worldwide.^1^ Unfortunately, 20%–30% of CRC patients are in the advanced stage, which cannot be easily rooted out through surgery. In addition, postoperative metastasis and recurrence severely affect patient survival.^2^ Currently, systemic radiotherapy and chemotherapy are the common intervention managements for preventing local tumor recurrence and distance metastasis after surgery. However, both radiotherapy and chemotherapy usually cause serious side effects and destroy immune systems, which limit their efficacy in prolonging patients' overall survival.^3^ Notably, surgery produces a transient immunosuppressive tumor microenvironment associated with wound healing, which leads to tumor cell proliferation, migration, invasion, and even immune escape.^4^ Therefore, it is urgent to develop new technologies to deal with postsurgery tumor recurrence and metastasis in CRC.

Cancer immunotherapy represented by immune checkpoint blockade (ICB) has held new promise for CRC treatment.^5^ For example, the anti‐programmed cell death receptor‐1 antibody (anti‐PD‐1 Ab) has been approved for high microsatellite instability (MSI‐H) or mismatch repair deficiency (d MMR) in advanced CRC.^6^ However, only approximately 5% of CRC patients have the characteristics of MSI‐H/d MMR. Most CRC patients have a low response rate to ICB, mainly because tumors lack immunogenicity and immunosuppressive microenvironment.^7^ Previous studies have found that combination therapy can improve the antitumor response and efficacy of ICB for CRC.^8^ In detail, some chemotherapy drugs, photodynamic therapy, and photothermal therapy (PTT) have a better synergistic effect with ICB.^9^, ^10^, ^11^

Photothermal therapy is a noninvasive means of cancer therapy, during which tumor tissues are ablated by the thermal effect of near‐infrared (NIR) light‐absorbing materials that can convert photon energy to heat to kill tumor cells.^12^ Compared to other traditional treatments, PTT has already showed plenty of merits such as non‐invasiveness, better controllability, and low incidence of complication.^13^ Furthermore, when combined with immunotherapy, PTT not only removes tumor tissues by thermal ablation but also induces tumor‐specific antigen release so as to activate the body's immune response, leading to inhibiting recurrence and metastasis.^14^, ^15^ Thus, a material‐based platform for local cancer treatment is a promising strategy, which could focus PTT on the surgical site, allow one to break local immune tolerance, and stimulate the systemic antitumor immune response, thus inhibiting the tumor recurrence/metastasis and avoiding system side effects.

Various materials with photothermal function have been reported and applied in PTT treatment.^16^ Given that most reported photothermal materials have not been verified in a clinic, U.S. Food and Drug Administration (FDA) approved indocyanine green (ICG), which possesses outstanding photothermal conversion efficiency upon NIR irradiation, as well as definite biocompatibility and biosafety, has received more expectations of clinical transformation.^17^ However, for local cancer treatment that focuses PTT on the surgical site, photothermal materials have to be easily applied to the surgical site and firmly fixed to the surgical site to ensure that they can play a photothermal function during the follow‐up treatment. While ICG is amphiphilic, the application of ICG alone as the photothermal reagent in PTT on the surgical site is almost impossible due to its water‐soluble nature and high diffusivity and rapid scavenging in vivo.^18^ Thus, there is a need to develop an ICG carrier, which can not only improve ICG retention and limit ICG diffusion but also realize convenient and accurate delivery of ICG onto the residual tumor site after the operation. Injectable hydrogels have received wide attention for their ability to carry photothermal components and applicability to complex and irregular organizational environment.^19^, ^20^, ^21^ However, it is very difficult to prevent amphiphilic ICG release from hydrogels that usually with an amphiphilic nature. In addition, in the humoral environment of most organs, hydrogel carriers lacking tissue adhesion performance will fall off from the surgical site, further limiting the curative effect.^22^

**FIGURE 1:**
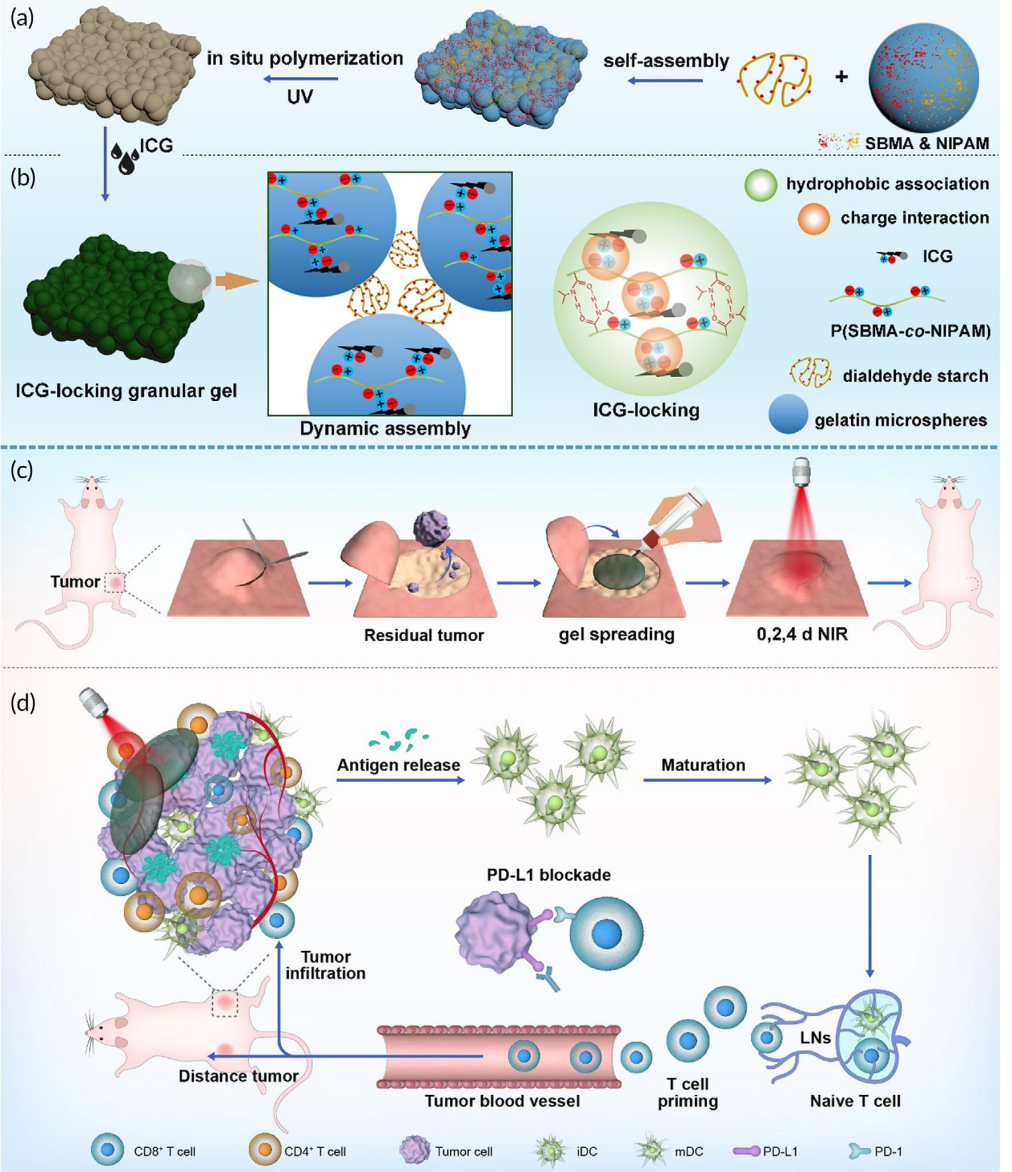
Illustration of granular hydrogel fabrication (a), structure (b), application (c), and therapy mechanism (d).

Thus, it is of high clinical value and also of great challenge to develop an injectable hydrogel with high ICG retention and tissue adhesive ability in the humoral environment to perform guaranteed PTT on the surgical site. Herein, according to the molecular structure of ICG with negatively charged hydrophilic side and hydrophobic side,^23^, ^24^ we designed an ICG‐locking injectable hydrogel based on gelatin microspheres/dialdehyde starch granular hydrogel compounded with poly(sulfobetaine methacrylate‐*co*‐N‐isopropylacrylamide) (P(SBMA‐*co*‐NIPAM)). The P(SBMA‐*co*‐NIPAM) that also possesses a negatively charged hydrophilic side and a hydrophobic side at body temperature was expected to effectively prevent ICG from releasing and diffusion. At the same time, the granular hydrogel was expected to be spread easily to the surgery site and adhere tightly to the surgery site for CRC postsurgical relapse and metastasis prevention. We hypothesized that such hydrogel could realize ICG application on the tumor site thus amplifying PTT efficiency. The combination of αPD‐L1 was expected to eliminate residual tumor cells and prevent tumor recurrence and metastasis by promoting both innate and adaptive antitumor immune responses (Figure 1).

## 2 EXPERIMENTAL SECTION

### 2.1 Materials

Dialdehyde starch, gelatin, sulfobetaine methacrylate (SBMA), indocyanine green (ICG), and 2‐Hydroxy‐4′‐(2‐hydroxyethoxy)‐2‐methylpropiophenone (I‐2959) were purchased from Aladdin (China). *N*‐Isopropyl acrylamide (NIPAM), *N*‐hydroxysuccinimide (NHS), 1‐(3‐Dimethylaminopropyl)‐3‐ethylcarbodiimide hydrochloride (EDC•HCl) was purchased from TCI (Japan). Fetal bovine serum (FBS) and penicillin‐streptomycin were obtained from Gibco BRL (Carlsbad, CA, USA). RPMI 1640 and phosphate‐buffered saline (PBS) were obtained from Hyclone (Thermo Fisher Scientific, Waltham, MA, USA). Cell Counting Kit‐8 (CCK‐8) and Annexin V‐FITC apoptosis detection kit were purchased from Dojindo (Kumamoto, Japan). In situ cell death detection kit, TMR red (TUNEL) was acquired from Roche (Roche Diagnostics GmbH, Germany). HMGB1 of human and mouse were purchased from Arigo Biolaboratories (Hsinchu City, Taiwan, ROC). ATP assay kit was acquired from Beyotime (Cat. S0026).

### 2.2 Preparation of gelatin microspheres

Gelatin (0.2 g) and NHS (0.117 g) were dissolved in 2 mL of deionized water at 50°C. EDC•HCl (0.228 g) was dissolved in 1 mL of deionized water. Span (2 mL) was mixed into petroleum ether (30 mL) and emulsified under 24,000 r/min for 30 s. The above gelatin/NHS solution and EDC•HCl solution were mixed evenly and quickly added into the emulsified petroleum ether (water oil ratio = 1:10) for further emulsification for 2 min, which was transferred into the round bottom flask containing 120 mL petroleum ether, and stirred at the stirring speed of 650 r/s. After 9 h, microspheres were obtained with three times ethanol washing and 24 h of vacuum drying.

Microspheres in dry and wet states were observed by scanning electron microscope (SEM) (SU‐1500, HITACHI) and stereomicroscope (S9EDI, Leica), respectively. Size statistics were achieved during stereomicroscope observation via its dimension measurement software.

### 2.3 Preparation of granular hydrogel

Dialdehyde starch was dispersed in deionized water at 70°C (concentration of 10 wt%), followed by being mixed with gelatin microspheres for centrifugation at 2000 rpm for 10 min. A granular hydrogel (GEL‐DAS) was obtained. A solution containing NIPAM, SBMA, and the photoinitiator LAP (total solid concentration of 10 wt%) was added into vacuum‐dried granular hydrogel (GEL‐DAS), which was then irradiated under ultraviolet light for in situ radical polymerization for 30 min and then dialyzed in deionized water to obtain the composite granular hydrogel (GEL‐DAS‐PSN). At the same time, a solution only contained SBMA and the photoinitiator LAP (total solid concentration of 10 wt%) was added into vacuum‐dried granular hydrogel (GEL‐DAS) and for polymerization under the same protocol to obtain the composite granular hydrogel (GEL‐DAS‐PS) as the control group. A solution only contained NIPAM and the photoinitiator LAP (total solid concentration of 10 wt%) was added into vacuum‐dried granular hydrogel (GEL‐DAS) and for polymerization under the same protocol to obtain the composite granular hydrogel (GEL‐DAS‐PN) as another control group.

For the rheological properties test, the granular hydrogels, GEL‐DAS and GEL‐DAS‐PSN were transferred to the fixture of the rheometer with a 12 mm flat plate clamp. The modulus‐frequency relationship was tested under the frequency scanning range of 0.01–100 Hz and the strain of 0.5%. Strain scanning measurements were carried out at a constant frequency of 1 Hz and a strain range from 0.1% to 1000%. The shear‐thinning behavior was studied by measuring the viscosity change with the shear rate increased from 0.001 to 100 s^−1^ continuously.^25^

For the swelling test, the vacuum‐dried granular hydrogels were weighted and immersed in deionized water at 25°C and weighed at 5, 10, 30, 60, 180, 300, 600, and 1800 s. The swelling ratio was calculated according to the following formula: SR = (W_x_‐W_0_)/W_0_, where W_x_ was the wet weight of the hydrogels at different time points, and W_0_ was the dry weight of the hydrogels.^25^

### 2.4 ICG retention in granular hydrogels

ICG solution was added into the granular hydrogels, GEL‐DAS, GEL‐DAS‐PSN, GEL‐DAS‐PN, and GEL‐DAS‐PS, and immersed in 0.01 mol/L PBS solution at 37°C for 24 h. Then, PBS solutions were collected into a cuvette, and evaluated on a Spectrophotometer (SpectraMax M2, Molecular Devices) at the wavelength of 660 nm. To determine the amount of ICG released from hydrogels, ICG adsorbed in hydrogels were immersed in PBS. At 1 day and 5 days, PBS was collected to measure the concentration of the released ICG via the UV–Vis absorption spectra to determine ICG release.^23^

### 2.5 Tissue adhesive force of granular hydrogels

The lap shear tests were employed to measure the tissue adhesion capability of granular hydrogels. The gelatin solution was spread onto glass slides and dried at 37°C. Granular hydrogels, GEL‐DAS and GEL‐DAS‐PSN were injected into the overlapped area between two glass slides at room temperature and compressed for 1 min. DXLL‐10000 tensile machine (D&G Measure Instruments, China) was used to carry out stretching at a shear velocity of 1.3 mm/min until two glass slides were wholly separated.^26^

### 2.6 In vitro cytotoxicity assays

HCT116 and CT26 were seeded into 96‐well plates separately at a density of 1 × 10^4^ cells per well and maintained with 100 μL RPMI 1640 medium. After 24 h, the culture media were replaced with 100 μL of fresh medium containing different concentrations of ICG and ICG@Hydrogel (CT26:0, 0.625, 1.25, 2.5, 5, 10, and 20 ug/mL; HCT116: 0, 0.625, 1.25, 2.5, 5, and 10 ug/mL). After 3 h, both of the above colorectal cells were washed by PBS and irradiated with or without 808 nm laser (1 W/cm^2^, MDL‐F‐808, Changchun New Industries Optoelectronics Technology Co., Ltd.) for 5 min, then changed into complete medium and incubated for another 24 h. The relative cell viability of the different groups was evaluated by the CCK‐8 assay (Dojindo).^27^

The cells were stained with PI (dead cells, red) and Calcein‐AM (live cells, green), respectively. The fluorescence images were obtained with an inverted fluorescence microscope (Leica).^27^

### 2.7 Flow cytometric cytotoxicity assay

CT26 and HCT116 (5 × 10^4^ cells/well) were seeded in 24‐well plates with 500 μL of culture medium overnight. Subsequently, the ICG or ICG@Hydrogel was added to the culture media containing different concentrations (CT26:0, 2.5, 5, 10, and 20 ug/mL; HCT116: 0, 1.25, 2.5, 5, and 10 ug/mL) for 3 h. After they were washed by PBS, the cells were exposed to 808 nm laser (1 W/cm2) for 5 min, then change into a complete medium. After incubation for 24 h, the cell suspension with a density of 1 × 10^6^/mL was diluted by binding buffer. Then, 5 μL annexin V‐FITC and 5 μL PI solution were added and incubated for 15 min in the dark, followed by an analysis with a flow cytometer (Beckman).^27^

### 2.8 In vitro photothermal characteristics

In vitro photothermal traits of the ICG@Hydrogel were characterized by monitoring the temperature change of ICG@Hydrogel aqueous solution under 808 nm laser (1 W/cm^2^) irradiation for 5 min. Besides, ICG@Hydrogel was just prepared by dissolving powder‐like hydrogel into 5 mg/mL ICG aqueous solution. Laser irradiation‐induced temperature elevation was recorded with an infrared imaging device (Fotric 111#L28).^12^

### 2.9 Immunogenic cell death (ICD)

HCT116 and CT26 cells (5 × 10^4^ cells/well) were seeded into 24‐well plates separately and maintained with 500 μL culture medium. After 24 h, the culture media were replaced with 500 μL of fresh medium containing different samples (PBS+L, ICG@Hydrogel, free ICG+L, ICG@Hydrogel+L). After 3 h later, cells were washed by PBS and irradiated with 808 nm laser (1 W/cm^2^) for 5 min, then change into a complete medium and incubated for another 24 h. The supernatants were collected to determine for Human or Mouse HMGB1 ELISA Kit, and cells were collected for ATP detection, according to the manufacturer's instructions.^11^

For the CRT exposure assay, the cells were seeded in 35‐mm confocal dishes at a density of 2 × 10^5^ cells/well for 22 h treated as described for the HMGB1 study. After incubation for 24 h, cells were fixed with 4% polyoxymethylene for 5 min, washed with PBS for 5 min × 3, permeabilized with 0.1% Triton X‐100 for 5 min, and washed with PBS for 5 min × 3. After being blocked with 5% fetal bovine serum in PBS for 1 h, followed by staining with Alexa Fluor 488‐CRT antibody (ab196158) for 4°C overnight, then washed with PBS three times and DAPI for 5 min and washed with PBS for 5 min × 3. Finally, the samples were observed with the Confocal Laser Scanning Microscope (CLSM 510; Carl Zeiss, Inc.).^11^

### 2.10 In vivo evaluation of hydrogel degradation and biocompatibility

All of the animal care, housing, and study procedures for mice were performed in accordance with the Guide for the Care and Use of Laboratory Animals and approved by Shanghai Putuo Hospital Ethics Committee (No. DWEC‐A‐202203005). The degradation of granular hydrogel in vivo was imaged by IVIS Spectrum In Vivo Imaging System (PerkinElmer IVIS Lumina III). BALB/c mice (6–8 weeks old) were subcutaneously coated with ICG@Hydrogel (ICG: 5 mg/mL) on the right flank during the surgery and then sutured. The mice were anesthetized with 2.5% pentobarbital sodium and imaged by IVIS to record the degradation of the granular hydrogel (ICG:Ex = 780 nm, Em = 831 nm)until the hydrogels disappeared.

To study the biocompatibility of the ICG@Hydrogel in vivo, the ICG@Hydrogel was inoculated to the flank of BALB/c mice. Mice were sacrificed weekly and took photos of the skin coated with ICG@Hydrogel.^19^

### 2.11 In vivo tumor models (antitumor, lung, and recurrence)

1 × 10^6^ of luciferase‐tagged CT26 cells in 100 μL of PBS were subcutaneously injected into the right flank of each female Balb/c mice (6–8 weeks old) to establish the tumor‐bearing mice model.^11^ When the tumor volume reached a volume of 100 mm^3^, mice were randomly divided into six groups (*n* = 6): (1) saline, (2) Hydrogel, (3) ICG@Hydrogel (ICG: 5 mg/mL), (4) αPD‐L1, (5) ICG@Hydrogel (ICG: 5 mg/mL)+L(Laser), (6) ICG@Hydrogel (ICG: 5 mg/mL)+L+αPD‐L1. Besides, mice were treated with tumor removal surgery in all groups, leaving about 1% residual tissue to mimic the residual microtumor in the real operation. Afterward, the wound was routinely coated with hydrogel for the hydrogel groups. Meantime, tumor tissue of the same size was inoculated on the left side to establish a bilateral mouse model, in order to test whether to eliminate the distant tumor. During the surgery, the mice were anesthetized with 2.5% pentobarbital sodium and sutured with sterile suture. For the laser groups, the entire wound region was irradiated at 0, 2, and 4 days (808 nm, 1 w/cm^2^) for 5 min after the operation. And for the αPD‐L1 groups, the mice were injected intravenously at 1, 3, and 5 days. Thermal imaging was captured using an infrared thermal imaging camera simultaneously. Tumor volume on both sides and weight of mice were measured every 2 days after the operation. Tumor volume was calculated using the following formula: tumor volume = 0.5 × length × width^2^.

For studies on the effect of PTT, when the tumor reached around 2500 mm^3^, all the mice were sacrificed. The lymph nodes, spleens, and tumor were harvested at the same time to detect immune cells. Simultaneously, the main organs including heart, liver, spleen, lung, and kidney were also collected for histology analysis.

For the tumor model of lung metastasis, the mice were intravenously infused with 2 × 10^6^ CT26 (Luc) tumor cells to generate a local tumor, the cells were washed with PBS before use and dissolved in 200 uL PBS. At 21 days after injection, imaged by IVIS to monitor local tumor and lung metastasis.

For studies on the tumor recurrence model, mice were randomly divided into four treatment groups (*n* = 6): (1) Ctrl, (2) αPD‐L1, (3) ICG@Hydrogel (ICG: 5 mg/mL)+L, (4) ICG@Hydrogel (ICG: 5 mg/mL)+L+αPD‐L1. The operation and treatment scheme were the same as the bilateral mouse model. CT26 cells were inoculated subcutaneously on the opposite side of the mouse on the 30th day after the operation, the mice were observed for 60 days after the operation, during which the tumor volume was recorded every 2 days, and the survival rate was also counted.

### 2.12 In vivo bioluminescence imaging

At 7, 14, 21, and 28 days after the operation, the tumor‐bearing mice were anesthetized with 2.5% pentobarbital sodium and injected with d‐luciferin (150 mg/kg), imaged by IVIS to monitor tumor and lung metastasis.^28^

### 2.13 In vivo photothermal characteristics(thermal image)

To examine the photothermal effect of the ICG@Hydrogel in vivo, the ICG concentration was 5 mg/kg, which was injected subcutaneously into the right blank of BALB/c mice. Then, the injection points were irradiated with 808 nm (1 W/cm^2^) laser for 5 min, and the temperature change was detected by an infrared imaging device.^12^

### 2.14 In vivo toxicity assays (organ HE and skin HE)

The hydrogel was applied to the right blank skin of mice. The mice were sacrificed on days 7, 14, 21, 28, and 36 postsurgery, and in situ hydrogel and surrounding skin tissue sections were retrieved from the surgical site. The major organs, including the heart, liver, spleen, lungs, kidneys, and tumors were also collected after the experiment finished, all these organizations were dissected, fixed in 10% formalin, embedded in paraffin, and sliced at 4 μm thickness. Then, slides were stained with hematoxylin and eosin (H&E) following a standard protocol and observed by a microscope (Leica).^11^

### 2.15 Cell flow cytometry analysis

Tumors, lymph nodes, and spleens were harvested, and the tissues were cut into small pieces with scissors and gently filtered through a 70 μm cell strainer. Red blood cells were removed with Red Blood Cell Lysis Buffer. Single‐cell suspensions were washed and resuspended with PBS. The cells were incubated with Fc‐Block (BioLegend) for 5 min on ice to avoid nonspecific binding. Subsequently, cells were stained with fluorophore‐conjugated antibodies in the dark for 30 min on ice.^11^

For analysis of T cells in tumors and spleens, cell suspensions were stained with CD3‐PE (BioLegend), CD4‐FITC (BD Pharmingen), and CD8a‐APC (BD Pharmingen).

For analysis of memory T cells in tumors, cells suspensions were stained with CD3‐PE (BioLegend), CD8‐PerCP‐Cy5.5 (BD Pharmingen), CD44‐FITC (Invitrogen), and CD62L‐APC (BD Pharmingen).

For analysis of Myeloid‐Derived Suppressor Cells (MDSCs) in spleens, cells suspensions were stained with CD11b‐PE (BioLegend), Gr‐1‐FITC (BioLegend), Ly‐6C‐APC (BD Pharmingen), and Ly‐6G‐PerCP‐Cy5.5 (BioLegend).

For analysis of Natural Killer cells (NK) in spleens, cell suspensions were stained with CD3‐PE (BioLegend) and CD335‐V450 (BD Horizon).

For analysis of mature DC cells in lymph nodes, cell suspensions were stained with CD11c‐PerCP‐Cy5.5 (BioLegend), CD80‐APC (BioLegend), and CD86‐FITC (Invitrogen). After washing, the cells are resuspended in the stain buffer and analyzed using flow cytometry (Beckman).

### 2.16 Immunohistochemistry assays

Tumors in different groups were collected, fixed in 10% formalin, and paraffin‐embedded tissue sections were performed. After blocking and permeabilization, tumor tissue sections were stained with Ki67 (ab15580) antibody, rinsed in PBS three times, and then incubated with secondary goat anti‐rabbit immunoglobulin G (IgG) at room temperature for 30 min. The slides were incubated with DAB (Abcam) for visualization after rinsing with PBS. Subsequently, the slides were washed in tap water, stained with hematoxylin for 15 s, rinsed in tap water again, and seal the slices.^11^

### 2.17 Immunofluorescence and TUNEL assays

For immunofluorescence staining, tumor sections were collected and permeated for 20 min, incubated with primary CD8 (ab217344) antibodies overnight at 4°C, followed by the secondary donkey anti‐rabbit IgG at room temperature for 1 h. DAPI was employed for nucleus staining for 5 min and sealing the slices. In the TUNEL assay, the slides were stained with the TUNEL technique on the in situ cell death detection kit according to the manufacturer's instructions. The fluorescent images were acquired on the CLSM.^11^

### 2.18 In vivo cytokine assay

Serum samples were collected from each group and TNF‐α (EMC102a), IFN‐γ (EBC101g), and IL‐6 (EMC004) were measured with ELISA kits according to the vendor's protocol.^29^

### 2.19 Statistical analysis

All data are presented as mean ± standard deviation (SD). Statistical analysis was performed using GraphPad Prism software version 8.01. To compare the difference between groups, the one‐way ANOVA test was utilized. T. Significant differences between the groups are expressed in the figures as *p < 0.05, **p < 0.01, and ***p < 0.001, respectively.

## 3 RESULTS

Using tissue adhesive hydrogel with photothermal function to cover surgical site for inhibiting postoperative recurrence and metastasis of CRC is a method with great potential to reduce mortality and morbidity. This study designed a granular hydrogel for carrying and trapping ICG with a photothermal function. The granular hydrogel was assembled from gelatin microgels by dialdehyde starch and interpenetrated with in situ co‐polymerized poly(sulfobetaine methacrylate‐*co*‐N‐isopropylacrylamide) (P(SBMA‐*co*‐NIPAM)). The main functions of the granular gel were tissue adhesion, ICG‐locking, and injectability. Hydrogel‐carrying ICG was to perform definite photothermal function and biosafety for photothermal therapy (PTT) combined with a checkpoint inhibitor.

### 3.1 Preparation and injectability of granular hydrogel

**FIGURE 2:**
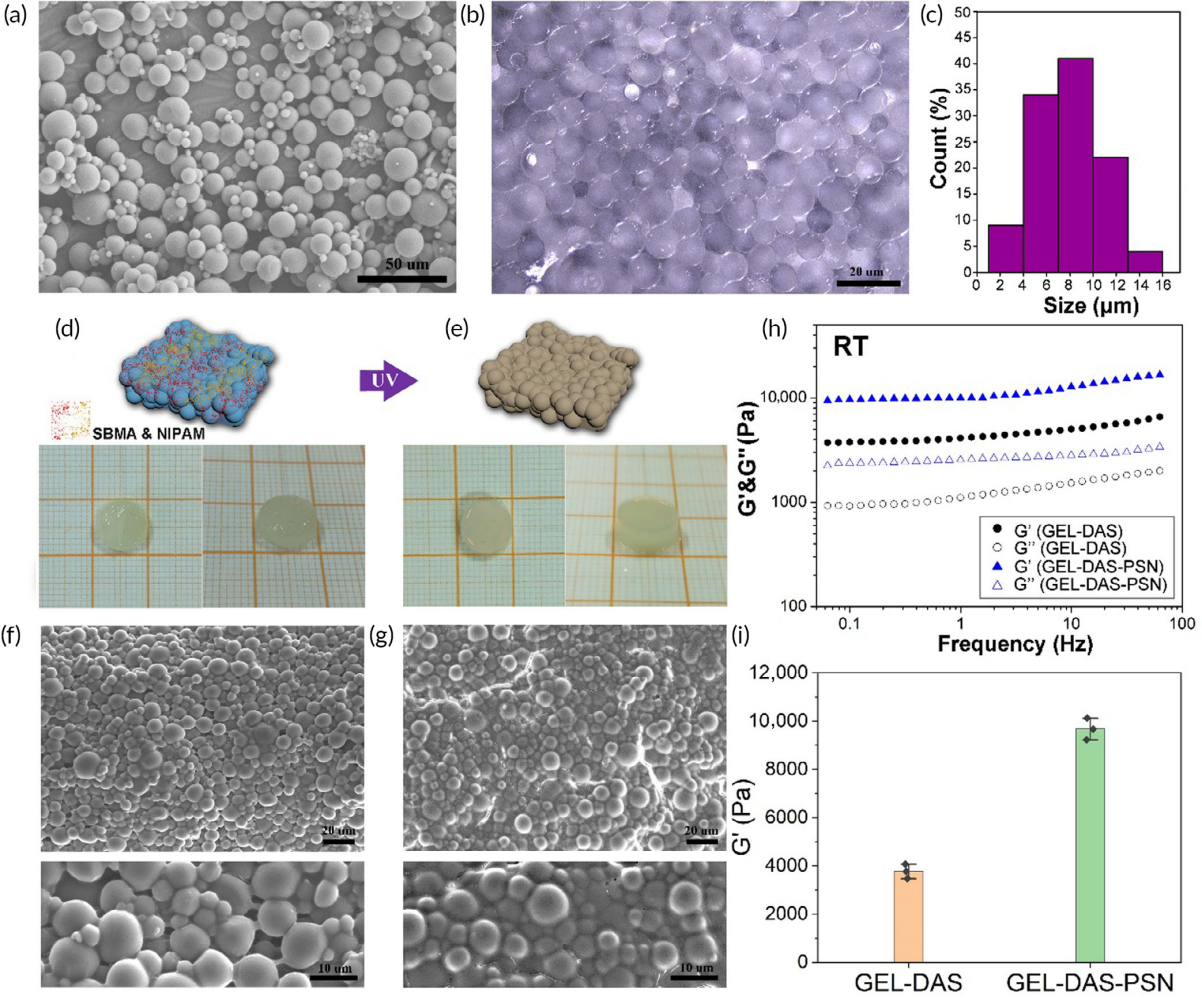
Fabrication of gelatin microspheres and granular hydrogels. (a) SEM image. (b) Wet microspheres observed by stereomicroscope. (c) Diameter statistics. (d) General observation of GEL‐DAS granular hydrogel. (e) General observation of GEL‐DAS‐PSN granular hydrogel. (f) SEM images of GEL‐DAS granular hydrogel. (g) SEM images of GEL‐DAS‐PSN granular hydrogel. (h) Storage modulus (G′) and loss modulus (G′) in the scanning range of 0.01–100 Hz (55°C, strain 0.5%). (i) Storage modulus (G′) comparison. *n* = 3, p* < 0.05.

In this study, gelatin microspheres were prepared by amidation cross‐linking combined with the emulsion method. There are abundant carboxyl and amino groups in gelatin molecules, which can be cross‐linked by amidation between these carboxyl and amino groups with the presence of EDC and NHS in the aqueous phase. Thus, cross‐linked gelatin microspheres by amide bonds were prepared in a water‐in‐oil emulsion system. According to the SEM image in Figure 2a, dry regular spherical particles were obtained. In water, the microspheres could adsorb water and swell with volume increase, forming microgels with a diameter range of 1–16 μm (Figure 2b,c).

Gelatin microspheres were assembled by dialdehyde starch. Dialdehyde starch contains a large number of aldehyde groups, which can form Schiff base interaction with gelatin.^30^ The dialdehyde starch suspension heated at 70°C and mixed with stacked gelatin microspheres could immediately form a bulk hydrogel as shown in Figure 2d. This hydrogel was named GEL‐DAS granular hydrogel. Subsequently, SBMA and NIPAM monomers in GEL‐DAS granular hydrogel co‐polymerized in situ to form poly (sulfobetaine methacrylate‐*co*‐*N*‐isopropylacrylamide) (P(SBMA‐*co*‐NIPAM)) that penetrating through GEL‐DAS granular hydrogel. The in situ co‐polymerized P(SBMA‐*co*‐NIPAM) inside GEL‐DAS granular hydrogel formed an interpenetrating network with gelatin microspheres, further physically cross‐linking the microspheres, forming composite granular hydrogel, GEL‐DAS‐PSN (Figure 2e). Of note, P(SBMA‐*co*‐NIPAM) was not the key to the formation of gelatin microspheres and GEL‐DAS‐PSN. The introduction of P(SBMA‐*co*‐NIPAM) did not significantly change the size of gelatin microspheres (Figure S1). Its functions, including ICG‐locking and adhesion strength promotion, were illustrated in the following evaluations. The temperature sensitivity of P(SBMA‐*co*‐NIPAM) was adjusted by changing the proportion of the two monomers (Table 1). The introduction of SBMA significantly raised the lower critical dissolution temperature (LCST) of the copolymer. The LCST of P3 was the closest to body temperature (37°C). At the same time, due to the lower LCST of P1 and P2, the requirements for injection ambient temperature of granular hydrogels that are made from P1 and P2 might be more stringent. Thus, P3 was used in the related studies. As shown in Figure 2f,g, both GEL‐DAS and GEL‐DAS‐PSN granular hydrogel were composed of assembled microspheres. However, the micro‐structure of the two granular hydrogels was different. Compared with GEL‐DAS, GEL‐DAS‐PSN granular hydrogel showed a denser assembly of gelatin microspheres.

**TABLE 1:** Synthesis of copolymer.

Sample	Monomer (mg)		Initiator (mg)	Solvent (mL)	Time (min)	LCST (°C)
	NIPAM	SBMA	LAP	H_2_O	UV	
P1	50	0	5	1	15	31
P2	40	10	5	1	15	34
P3	35	15	5	1	15	37
P4	25	25	5	1	15	45

There were multi‐networks in GEL‐DAS‐PSN granular hydrogel, including a chemical cross‐linking network of gelatin microspheres, Schiff Base interaction between dialdehyde starch and gelatin microspheres, and interpenetrating network among P(SBMA‐*co*‐NIPAM), as well as hydrophobic aggregation of PNIPAM at body temperature. For one thing, as shown in Figure 2h, the storage modulus (G′) was greater than the loss modulus (G″) under the scanning range of 0.01 to 100 Hz (25°C, strain 0.1%), illustrating that both GEL‐DAS and GEL‐DAS‐PSN granular hydrogel exhibited elasticity of solid hydrogel. In addition, due to the addition of P(SBMA‐*co*‐NIPAM), the G′ of GEL‐DAS‐PSN granular hydrogel was significantly higher than that of GEL‐DAS granular hydrogel (Figure 2i).

**FIGURE 3:**
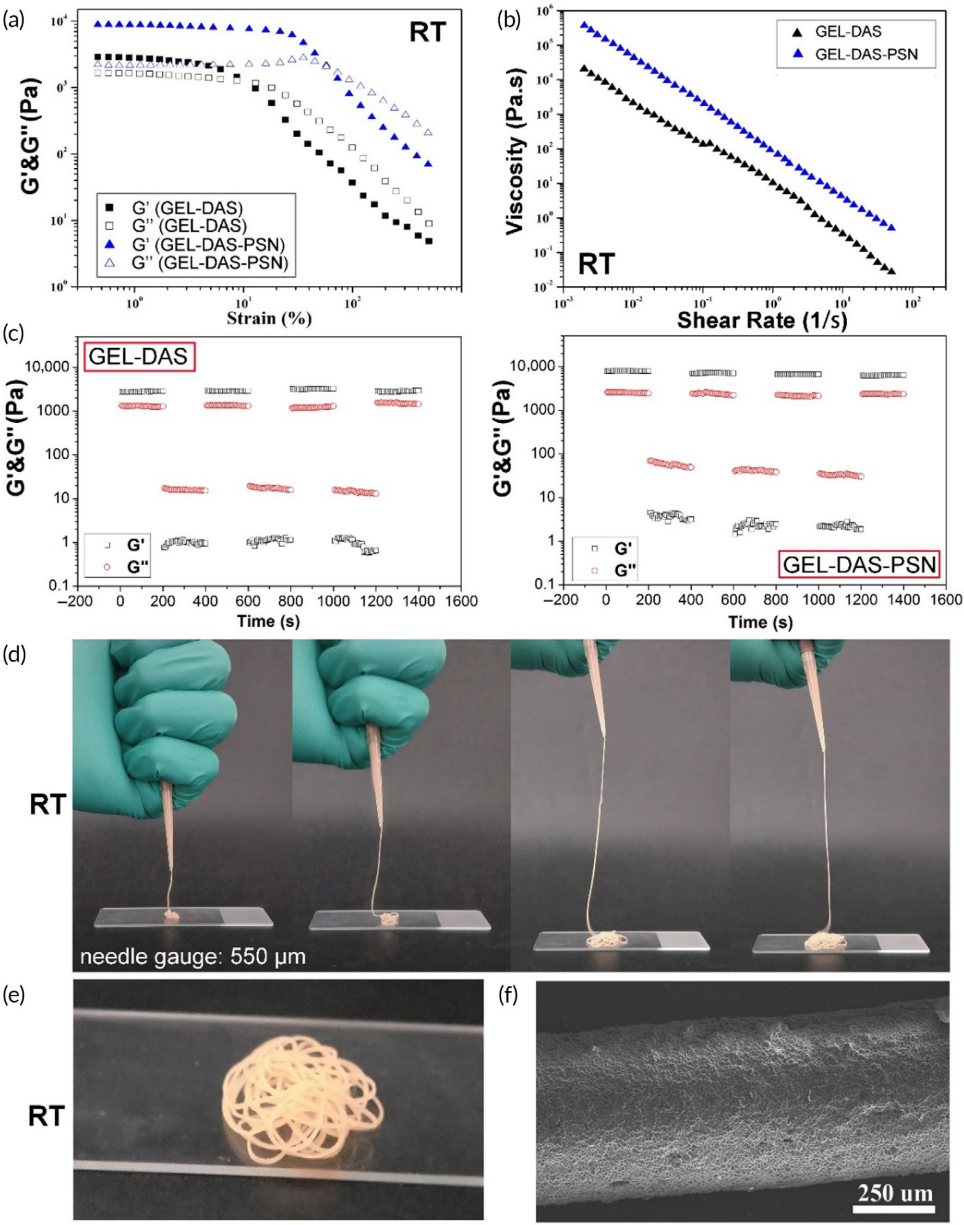
Injectability of granular hydrogels. (a) Storage modulus (G′) and loss modulus (G″) with strain scanning (0.01%–1000%, 1 Hz). (b) Shear viscosity with increasing shear rates (0.001–100 s^− 1^) of granular hydrogel. (c) Evaluation of self‐recovery of the granular hydrogels under alternating strains of 1% and 500%. (d) General observation of injection of GEL‐DAS‐PSN granular hydrogel at room temperature. (e) General observation of extruded hydrogel filament. (f) SEM image of the filament.

For another, the reversible Schiff Base interaction also realized the injectability of granular hydrogels at a temperature lower than 37°C. As shown in Figure 3a, when the strain was small, G′ was higher than G″. When the strain increased, G′ began to decline, while G″ began to increase to intersect with G′. The intersection point indicated the yield strain of hydrogel. When the yield strain was exceeded, G″ was greater than G′, indicating that the granular hydrogels showed viscous behavior. Of note, the yield strain of GEL‐DAS‐PSN granular hydrogel was higher than that of GEL‐DAS granular hydrogel. In addition, the viscosity of hydrogel showed a straight downward as the shear rate increased from 0.001 to 100 s^− 1^. The granular hydrogels changed from the solid state to the “liquid state” with low viscosity, revealing the flow ability of the granular hydrogel during injection (Figure 3b). Besides, Figure 3c showed that the granular gels maintained their moduli after cyclic low‐high strain action. Under low strain, G′ was greater than the G″. When the strain increased to 500%, the hydrogel was damaged, G′ decreased quickly and was lower than G″. When the strain returned to the initial 1%, G′ immediately recovered without significant decrease, indicating the self‐healing property of the granular gels. The introduction of P(SBMA‐*co*‐NIPAM) did not show a significant effect on gel recovery.

Due to the shear‐thinning, the solid granular hydrogel could evenly flow from nozzle and recovered to the solid state immediately after extrusion, forming a regular filament that extended vertically without breaking at room temperature (Figure 3d). The filament showed even and regular morphology without extravasation. After extrusion, as shown in Figure 3e, the filament was bent and stacked with no collapse on the surface glass surface. According to SEM observation (Figure 3f), the filament was composed of densely assembled microspheres. While GEL‐DAS‐PSN granular hydrogel could not be extruded when the temperature was higher than 37°C because of the gelation of P(SBMA‐*co*‐NIPAM). This was one of the reasons why P3 was chosen in the following studies.

### 3.2 ICG retention in granular hydrogel

**FIGURE 4:**
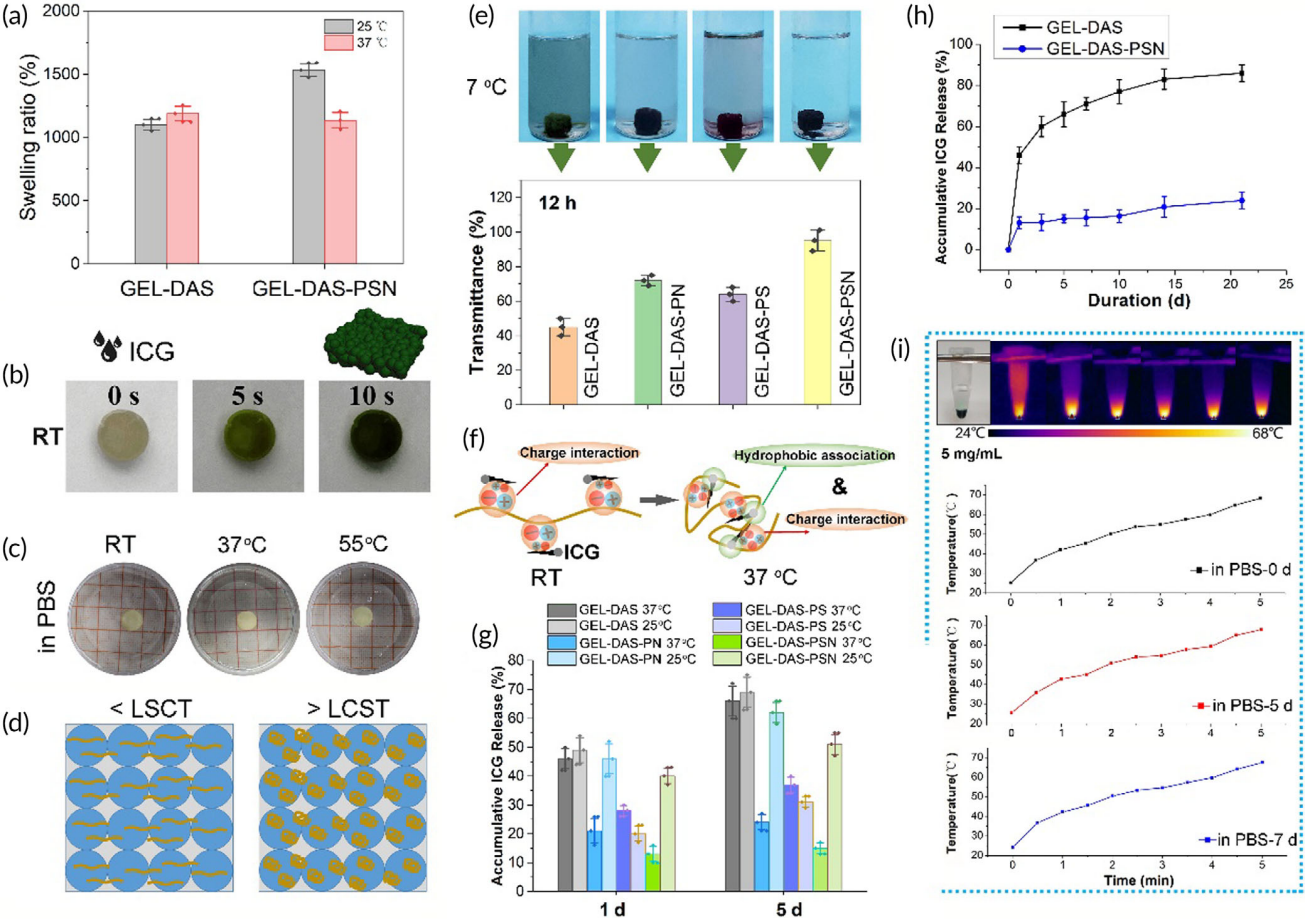
ICG locking in the granular hydrogel. (a) Swelling behavior of granular hydrogels. (b) General observation of quick adsorption of ICG into granular hydrogel. (c) General observation of granular hydrogel at different temperatures. (d) Diagram to show volume stability. (e) General observation of ICG granular gels in PBS to observe ICG retention and transmittance of the PBS at 12 h. (f) Diagram to show ICG‐locking mechanism. (g) ICG release from different granular hydrogels at 37°C and 25°C. (h) ICG release curves from hydrogel GEL‐DAS and GEL‐DAS‐PSN. (i) Photothermal images of ICG‐locking gel in PBS at different time points with the increase of laser irradiation time and the quantitative data. *n* = 3, *p < 0.05.

The GEL‐DAS‐PSN granular hydrogel was then employed as a carrier to combine with ICG for preparing ICG granular hydrogel. As shown in Figure 4a, the swelling behavior showed that both GEL‐DAS and GEL‐DAS‐PSN exhibited quick adsorption of water after being immersed in PBS at room temperature. The swelling ratio quickly reached over 600 wt% within 60 s. Besides, the equilibrium swelling degree of GEL‐DAS‐PSN granular hydrogel was higher than that of GEL‐DAS granular hydrogel. At the same time, due to the presence of P(SBMA‐*co*‐NIPAM), the equilibrium swelling degree of GEL‐DAS‐PSN granular hydrogel at 37°C was reduced. Thus, one of the unique features of the granular hydrogel was that it could quickly adsorb aqueous solution, such as ICG solution. As shown in Figure 4b, the granular hydrogel adsorbed the ICG solution immediately after ICG dropping, forming a granular hydrogel with dark green color within 10 s.

P(SBMA‐*co*‐NIPAM) in granular hydrogel played two important functions. On the one hand, a PNIPAM‐based copolymer could form stable physical hydrogel at body temperature. Thus, P(SBMA‐*co*‐NIPAM) could further stabilize the granular hydrogel at body temperature in vivo. Of note, PNIPAM‐based polymers are a kind of polymers that possess volume phase transition.^31^ When the temperature is higher than its low critical dissolution temperature, the molecular chains curl up and gather through hydrophobic interaction, resulting in network collapse and volume shrinkage. The higher the temperature, the more obvious the volume shrinkage. However, According to Figure 4c, the volume shrinkage was not found significantly in the present granular hydrogel. This might be because the granular hydrogel was constructed by densely assembled gelatin microgels, which mainly determined the volume of the hydrogel. Although the interpenetrating PNIPAM‐based copolymers collapsed at 37°C, their collapse was not strong enough to lead to volume shrinkage of gelatin microspheres. Thus the granular hydrogel showed no significant volume change at 37°C and even 55°C (the maximum temperature for PTT in this study). This was important for adequate coverage on the surgical site and also could avoid explosive release of ICG caused by sharp volume shrinkage.

On the other hand, the presence of P(SBMA‐*co*‐NIPAM) copolymer could significantly enhance the ICG retention in granular hydrogel via molecular chain collapse and charge interaction. Thus, hydrophobic association and charge interaction could be used to lock ICG in the hydrogel. As shown in Figure 4e, granular hydrogels containing ICG were immersed in PBS at 37°C for 12 h. Granular hydrogel without P(SBMA‐*co*‐NIPAM), (GEL‐DAS) showed significant release of ICG to PBS. While PBS that immersed with granular hydrogel with P(SBMA‐*co*‐NIPAM) copolymer was more clear and transparent, indicating the effective retention of ICG in hydrogel. To illustrate the locking effect, this study fabricated granular hydrogels containing PNIPAM homopolymer (GEL‐DAS‐PN) and PSBMA homopolymer (GEL‐DAS‐PS). The introduction of either PNIPAM or PSBMA was found to promote the retention of ICG at 37°C. However, ICG retention in GEL‐DAS‐PS granular hydrogel was weaker than that in GEL‐DAS‐PN and GEL‐DAS‐PSN, indicating that PNIPAM in copolymer was more effective to prevent the release of ICG at body temperature. While PBS that immersed with GEL‐DAS‐PSN was the clearest and the most transparent, revealing the ICG retention was further enhanced with the synergistic effect of PNIPAM and PSBMA.

Accordingly, ICG is amphiphilic in nature, possessing a hydrophobic side and a negatively charged hydrophilic side.^23^, ^24^ PSBMA fragments in the P(SBMA‐*co*‐NIPAM) copolymer could bind ICG via charge interaction. In addition, at 37°C, PNIPAM fragments in copolymer collapse through hydrophobicity, forming aggregations with ICG via hydrophobic association (Figure 4f). To confirm the possible mechanism, the accumulative release of ICG at 1 day and 5 days was monitored at both 25 and 37°C. According to Figure 4g, at 37°C, GEL‐DAS hydrogel showed the highest amount of released ICG, while GEL‐DAS‐PSN hydrogel showed the lowest amount of released ICG. The total release of ICG from GEL‐DAS‐PSN hydrogel at 5 days was similar to that at 1 day, revealing that P(SBMA‐*co*‐NIPAM) copolymer in hydrogel held significant ICG locking. Compared with GEL‐DAS‐PS hydrogel, GEL‐DAS‐PN showed reduced ICG release. The released result at 37°C was consistent with the diaphaneity test result. Of note, ICG release from GEL‐DAS‐PN, GEL‐DAS‐PS, and GEL‐DAS‐PSN hydrogels at 25°C was different from that at 37°C. ICG showed low release from GEL‐DAS‐PN at 37°C, while it showed high release at 25°C. This was related to the extension of the coiled molecular chain at lower temperature, resulting in the disappearance of hydrophobic aggregation, leading to faster release of ICG. ICG release from GEL‐DAS‐PSN hydrogel at 25°C showed a similar result, revealing that the hydrophobic association of ICG with PNIPAM was one of the ICG‐locking mechanism. ICG release from GEL‐DAS‐PS hydrogel at 25°C and 37°C was lower than that from GEL‐DAS hydrogel, illustrating the effect of charge interaction on ICG‐locking. Thus, the synergistic effect of charge interaction and hydrophobic association realized the tight bond of ICG to the GEL‐DAS‐PSN, so as to guarantee the continuous photothermal effect during the treatment period. The long‐term in vitro (Figure 4h) and in vivo (Figure S3) ICG release from GEL‐DAS hydrogel and GEL‐DAS‐PSN hydrogel was also monitored. As shown in Figure 4h, Only 20% of ICG in total was released from GEL‐DAS‐PSN hydrogel, leaving nearly 80% of ICG locked in hydrogel, indicating that ICG release from GEL‐DAS‐PSN hydrogel was significantly limited when compared to that from GEL‐DAS hydrogel, ensuring the application of photothermal performance in the treatment scheme. Besides, the in vivo ICG release from GEL‐DAS hydrogel was relatively faster in 3 weeks when compared to that from GEL‐DAS‐PSN hydrogel, indicating the tight bond of ICG to the GEL‐DAS‐PSN (Figure S3). These results were consistent with our in vitro study (Figure 4h).

Thus, the photothermal conversion efficiency activity of ICG granular gel was evaluated by measuring the temperature changes under 808 nm radiation. As expected, the temperature increased rapidly along with time within 5 min then remained at 68°C (Figure 4I). Moreover, during in vivo treatment, there were three times of irradiation within 7 days. Thus, ICG granular gel immersed in PBS in vitro for 5 and 7 days was also subjected to photothermal conversion evaluation. As shown in Figure 4i, the ICG granular gel kept its photothermal conversion efficiency without significant change, indicating that ICG granular gel possessed guaranteed photothermal conversion property for in vivo treatment. The GEL‐DAS‐PSN granular hydrogel carrying and retaining ICG was thus defined as ICG‐locking gel.

### 3.3 Tissue adhesion performance of ICG‐locking gel

**FIGURE 5:**
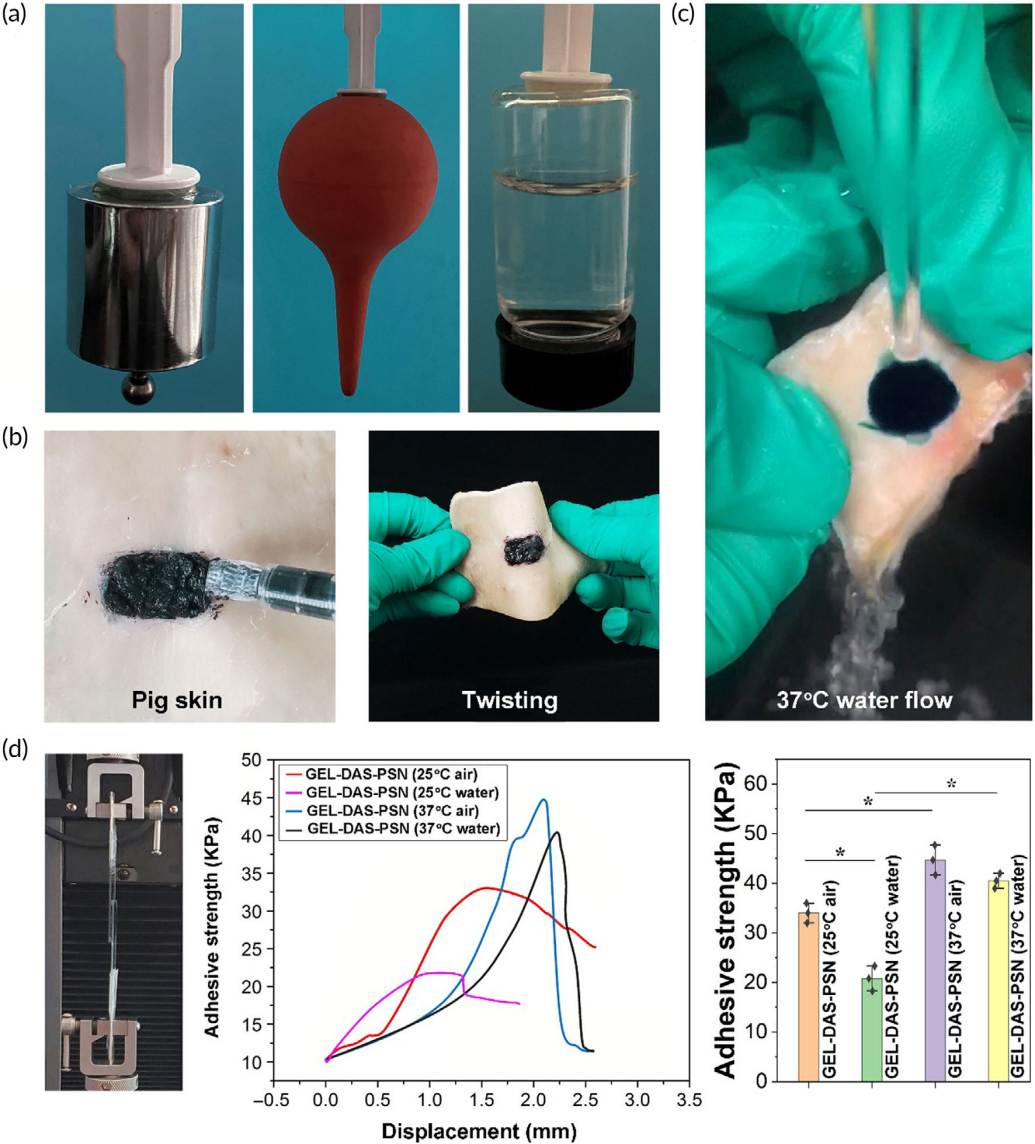
Tissue adhesive ability of ICG granular gel. (a) General observation of granular gel adhered on different matrixes, including plastic, metal, rubber, and glass in the air at room temperature. (b) and (c) General observation of ICG‐locking gel spread and attached to pig skin. (d) Lap shear tests and displacement‐adhesive strength curves. *n* = 3, *p < 0.05.

Due to the presence of dialdehyde starch, the granular gel exhibited significant tissue adhesive ability. As shown in Figure 5a, the GEL‐DAS‐PSN granular hydrogel could adhere to different matrices, including plastic, metal, rubber, and glass, which could bear a certain load in the air at room temperature. During in vivo application, dialdehyde starch could provide abundant hydrogen bonding and Schiff Base bonding with tissues to realize tissue attachment.^32^, ^33^ As shown in Figure 5b, the granular hydrogel containing ICG could be extruded and spread onto pig skin‐like creams. Of note, the ICG‐locking gel adhered tightly to the pig skin even when being rinsed with warm water (Figure 5c).

Then, the ICG‐locking gel was injected into glasses covered with gelatin to evaluate its tissue adhesive ability at body temperature. As shown in Figure 5d, two glasses coated with gelatin were adhered with ICG‐locking gel. The lap shear tests illustrated that GEL‐DAS‐PSN showed larger displacement and higher adhesive strength at 37°C. According to Figure S2, granular hydrogel, GEL‐DAS without P(SBMA‐*co*‐NIPAM) copolymer also showed high adhesive strength, while gelatin microspheres with P(SBMA‐*co*‐NIPAM) copolymer without dialdehyde starch showed significantly lower adhesive strength, indicating the important contribution of dialdehyde starch to hydrogel adhesion. Of note, in water, the adhesive strength of GEL‐DAS significantly reduced, while in water at 37°C, the adhesive strength of GEL‐DAS‐PSN did not reduce, and was higher than that at room temperature. This might be related to the thermosensitivity of the P(SBMA‐*co*‐NIPAM) copolymer. At 37°C, the P(SBMA‐*co*‐NIPAM) gelled to further enhance the granular hydrogel, so as to improve the adhesive force. At the same time, the P(SBMA‐*co*‐NIPAM) showed hydrophobic characteristics to prevent excessive water molecules from destroying the adhesion between gel and tissue.

Thus, gelatin and dialdehyde starch contributed the most to construction of granular hydrogel and its tissue adhesion ability, while the introduction of P(SBMA‐*co*‐NIPAM) copolymer not only realized the ICG retention in hydrogel but also ensured sufficient adhesion of the hydrogel in a wet environment.

### 3.4 In vivo biodegradation of granular hydrogel

**FIGURE 6:**
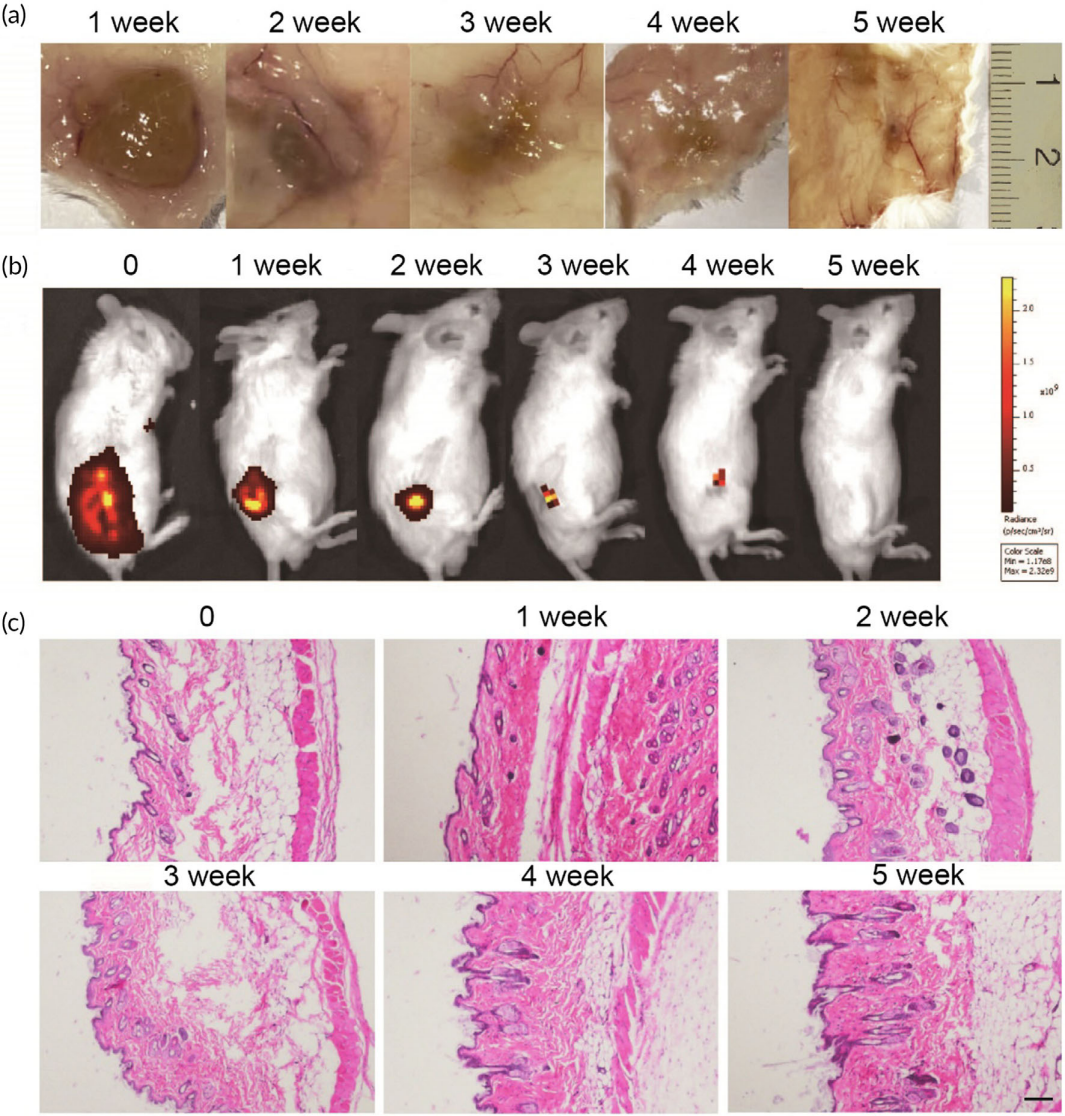
In vivo gelation and biodegradation of hydrogels. (a) In vivo biodegradation pattern of GEL‐DAS‐PSN granular gel over time. (b) In vivo extended release of ICG‐locking gel recorded by IVIS. (c) Representative histological examinations of the skins with H&E stainings (bar scale: 100 μm).

**FIGURE 7:**
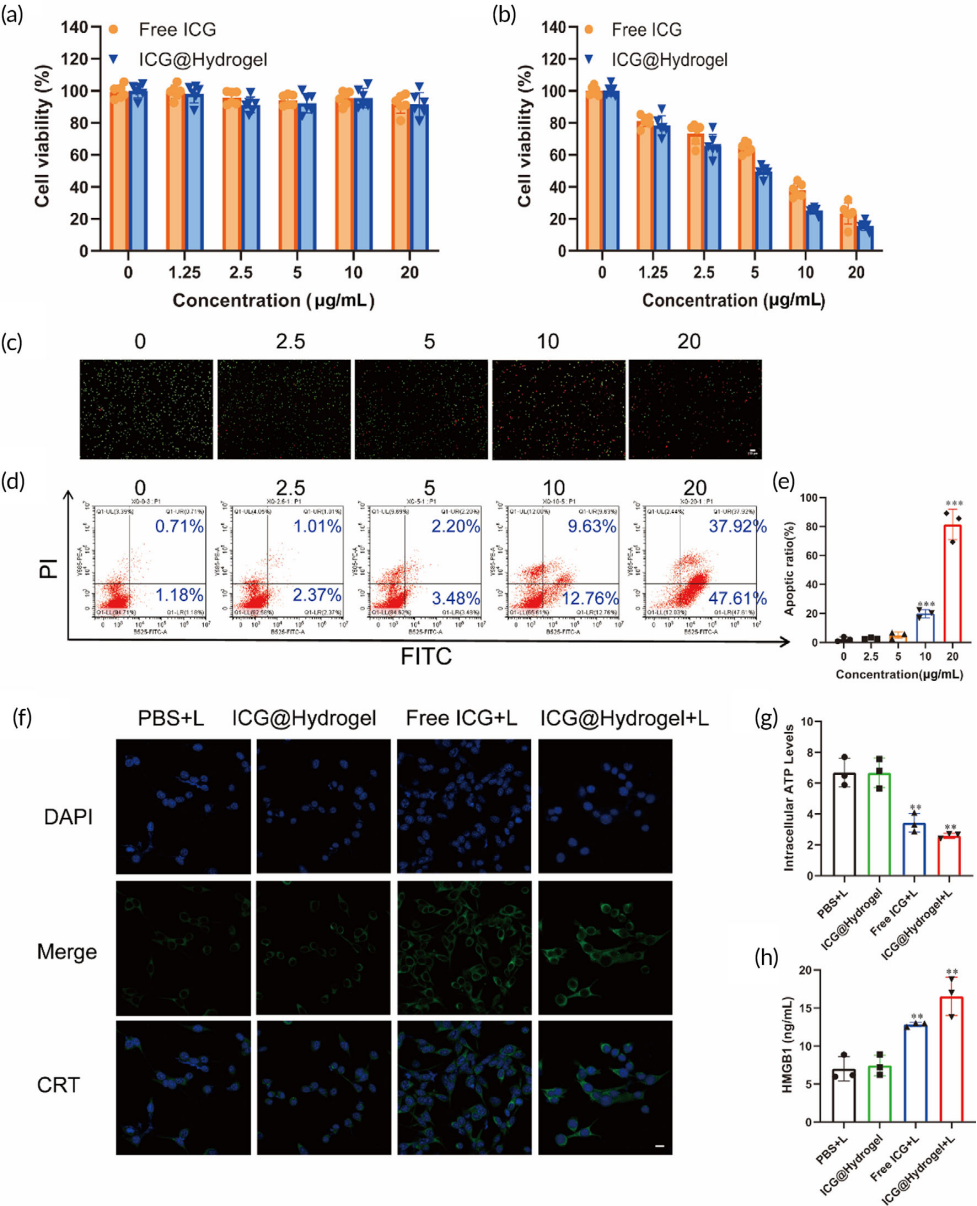
The antitumor effects of ICG‐locking gel and evaluations of ICD in vitro. Cell viability of CT26 cells after treatment with free ICG solution and ICG‐locking gel (a) without or (b) with NIR irradiation (808 nm, 1 W/cm^2^) for 24 h determined by CCK‐8 assay. (c) Calcein‐AM/PI double staining of CT26 cells treated with different concentrations of ICG‐locking gel with NIR irradiation (808 nm, 1 W/cm^2^) (bar scale: 100 μm). (d) Apoptosis analysis of CT26 cells resulting from combined treatment with different concentrations of ICG‐locking gel and NIR irradiation (808 nm, 1 W/cm^2^) for 24 h. (e) Quantification for the apoptosis rate of CT26 cells. (f) CLSM images of CRT exposure on the surface of CT26 cells after different treatments with ICG‐locking gel and NIR irradiation (808 nm, 1 W/cm^2^) for 24 h (bar scale: 20 μm). (g, h) Extracellular HMGB1 and intracellular ATP levels of CT26 cells after different treatments as indicated. Data are presented as the mean ± SD (*n* = 3) for (e, g, h). (PBS+L = PBS and irradiation treatment; ICG@Hydrogel = ICG‐locking gel treatment; Free ICG+L = ICG and irradiation treatment; ICG@Hydrogel+L = ICG‐locking gel and irradiation treatment). (*p < 0.05, **p < 0.05, and ***p < 0.001).

The biodegradation and biocompatibility properties of implanted hydrogels are critical for clinical applications.^30^ At first, the degradation of GEL‐DAS‐PSN hydrogel was estimated in vivo. The hydrogels were placed through extrusion subcutaneously into the flank of Balb/c mouse. The nodules that formed right after implantation were collected and photographed at different time points. As shown in Figure 6a, the hydrogels gradually degraded and disappeared completely in 5 weeks. At the same time, The degradation of ICG‐locking gel was monitored by fluorescence in vivo imaging system (IVIS) imaging, and it was noticed that total degradation was achieved at 5 weeks, which was consistent with the above result (Figure 6b). The addition of ICG had no influence on the degradation of the hydrogel. It was vital that the surrounding tissue at the implantation site returned to normal. In addition, no skin damage and toxicity were observed during the test period by hematoxylin and eosin (H&E) (Figure 6c). The in vitro cytotoxicity assay was further determined to confirm the biosafety of hydrogels. No obvious toxicity was observed both in CT26 and HCT116 cells (Figure 7a, Figure S4). These results indicated that the granular gel had good biocompatibility in vivo and in vitro.

### 3.5 In vitro‐induced ICD

The CCK‐8 assay was used to investigate the cytotoxicity in HCT116 and CT26 cells treated with different concentrations of ICG‐locking gel with 808 nm laser irradiation. The viability of the cells decreased with increasing concentration of ICG‐locking gel under light exposure concentration (Figure 7b, Figure S5). The IC50 value was calculated to be 6.68 ± 0.89 μg/mL and 6.80 ± 1.19 μg/mL for the HCT116 cells and 9.80 ± 2.67 μg/mL and 6.26 ± 1.61 μg/mL for the CT26 cells, respectively.

In addition, the cell viability of HCT116 and CT26 cells treated with ICG‐locking gel with 808 nm laser irradiation was also performed by calcein‐AM/PI costaining (Figure 7c, Figure S6). The results were in good agreement with the CCK‐8 assay. Subsequently, in order to evaluate the cell apoptosis caused by ICG‐locking gel under the laser, the flow cytometry analysis was examined by Annexin V‐FITC/PI apoptosis detection kit. As shown in Figure 7d, e and Figure S7, ICG‐locking gel with 808 nm laser irradiation could effectively induce dose‐dependent cell apoptosis. Taken together, ICG laser irradiation possessed superior antitumor PTT activity.

ICD is known to recruit antigen‐presenting cells, dendritic cells (DCs), and promote them to engulf tumor antigen, then activate their maturation.^31^ ICD is characterized by the release of DAMPs, including CRT, HMGB1, and ATP.^34^ Given that PTT could induce ICD in tumor cells, we further explored whether ICG‐locking gel‐mediated PTT could exert an effect on CRT, ATP, and HMGB1. As indicated in Figure 7f and Figure S8, ICG‐locking gel with irradiation‐induced excellent CRT exposure according to the immunofluorescence in CT26 cells. Meanwhile, the extracellular HMGB1 concentration increased while the intracellular ATP level decreased significantly upon ICG‐locking gel treatment by ELISA assay (Figure 7g, h, Figure S9). To further investigate whether ICG‐locking gel‐mediated PTT‐induced DCs maturation, a transwell system was employed. The upper chamber containing CT26 cells was pretreated with PBS+laser, ICG@Hydrogel, ICG+laser, or ICG@Hydrogel+laser, and the lower chamber was seeded with DCs harvested from BALB/c mice for 24 h. We then used flow cytometry to detect the expression of CD11c, CD80, and CD86, which represents mature DCs. As shown in Figure S10, the ICG+laser group showed a higher level of DCs maturation (72.23%) than the PBS+laser treatment (56.28%) and the ICG@Hydrogel (60.46%) treatment. And the ICG@Hydrogel plus a laser group led to the highest level of DCs maturation (81.19%). Our findings suggested that ICG‐locking gel‐mediated PTT could effectively induce ICD in CRC cells, which might hold excellent potential in antitumor immunotherapy.

### 3.6 Inhibition of postsurgery tumor local recurrence and distant metastasis in vivo

**FIGURE 8:**
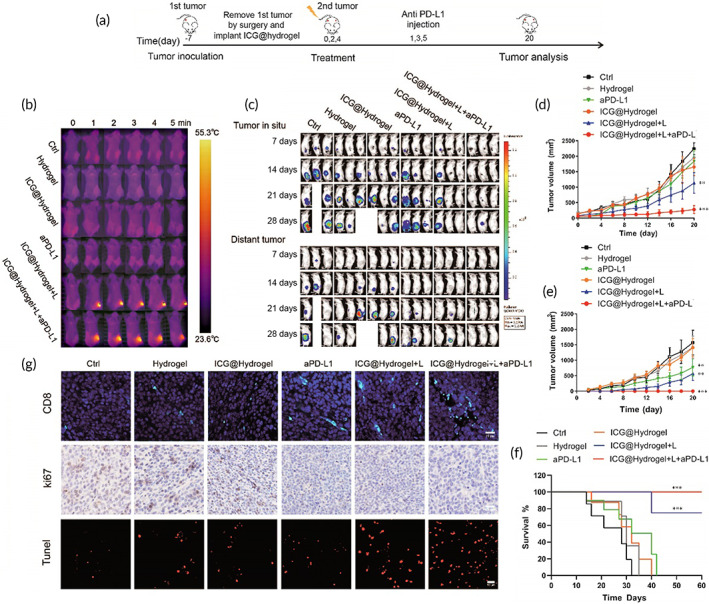
The antitumor and abscopal effect in vivo. (a) Schedule of tumor inoculation and treatment of bilateral tumor model. (b) Photothermal images of BALB/c tumor‐bearing mice exposed to NIR laser (808 nm, 1 W/cm^2^) after various treatments. (c) In vivo bioluminescence images to evaluate the growth of recurrent and distal tumors after various treatments. (d), (e) Growth curves of primary tumors and distant tumors over the course of 20 days after various treatments. (f) Survival percentages in the corresponding treatment groups described in (c), *p*<0.001 versus Ctrl. (g) Representative images of Ki67 staining, TUNEL staining, and CD8 immunofluorescence staining of tumors from different groups (bar scale: 50 μm). Data are presented as the mean ± SD (*n* = 6) for (d–f). (Ctrl = no treatment; Hydrogel = granular hydrogel without ICG treatment; ICG@Hydrogel = ICG‐locking gel treatment; αPD‐L1 = αPD‐L1 treatment; ICG@Hydrogel+L = ICG‐locking gel and irradiation treatment; ICG@Hydrogel+L+αPD‐L1 = ICG‐locking gel and irradiation treatment combined with αPD‐L1). (*p < 0.05, **p < 0.05, and ***p < 0.001).

Immune checkpoint inhibitors have been approved for the treatment of metastatic MSI‐H/d MMR CRC.^35^ The antitumor effect of the ICG‐locking gel‐mediated PTT combined with αPD‐L1 on CRC was further investigated in a bilateral tumor model. In order to simulate the clinical situation that some tumors could not be removed completely by surgery, especially for advanced‐stage cancers, we performed the surgery as illustrated in Figure 8a. In brief, 1 × 10^7^ CT26 cells were subcutaneously inoculated into the right flank as primary tumors and 2 × 10^6^ cells into the left flank to mimic metastases. 7 days later, 90% primary tumor was surgically removed, leaving 10% residual tumor to establish a postoperative model. Then, the ICG‐locking gel was coated into the tumor resection cavity and received NIR irradiation (808 nm, 1 W/cm^2^) for 5 min. As indicated in Figure 8b, the temperature of ICG@Hydrogel+L group (ICG‐locking gel and irradiation treatment) quickly rose to 55.3°C within 2 min and then remained around 55.0°C during irradiation. However, the temperature of the hydrogel group (granular gel without ICG treatment) and αPD‐L1 group (αPD‐L1 treatment) had no significant difference with the Ctrl group (control group), which suggested that the ICG‐locking gel showed a sufficient photothermal conversion property in vivo.

We verified the effect of ICG‐locking gel‐mediated PTT combined with αPD‐L1 on recurrence after colon cancer resection. As shown in the bioluminescent IVIS imaging and tumor growth curves, the ICG@Hydrogel+L+αPD‐L1 group (ICG‐locking gel and irradiation treatment combined with αPD‐L1) most effectively prevented local tumor recurrence and distance metastasis, while Hydrogel group (granular gel without ICG treatment), ICG@Hydrogel group (ICG‐locking gel treatment), and αPD‐L1 group (αPD‐L1 treatment) showed a limited effect in inhibiting the tumor growth on both sides (Figure 8c). Owing to the PTT‐elicited antitumor immune responses enhanced by ICG‐locking gel, the treatment in the ICG@Hydrogel+L group (ICG‐locking gel and irradiation treatment) resulted in the elimination of four out of six local tumors on the right side, and obviously delayed growth of distant tumors on the left sides. Importantly, the efficacy was further amplified by αPD‐L1, as indicated by the elimination of five out of six local tumors on the right side, and the elimination of all distant tumors on the left side. Meanwhile, we counted the survival rate within 60 days of mice in different groups (Figure 8d, e), ICG@Hydrogel+L+αPD‐L1 group showed a remarkable extension of survival time (more than 60 days) when compared to other groups (Figure 8f).

The tumor cell proliferation of tumor sections was evaluated by Immunohistochemical Ki67 staining. As indicated in Figure 8g, the combination of ICG‐locking gel and αPD‐L1 exhibited much lower Ki67 expression, which indicated markedly decreased tumor cell proliferation after treatment. In TUNEL‐stained tumor sections, the highest apoptotic rate was also detected in the combination group. In addition, there was no visible body weight loss among different groups, which was shown in Figure S11A. According to H&E staining results, no noticeable damage was observed in the major organs of mice (Figure S11B). These results confirmed that ICG‐locking gel had no obvious systemic toxicity.

**FIGURE 9:**
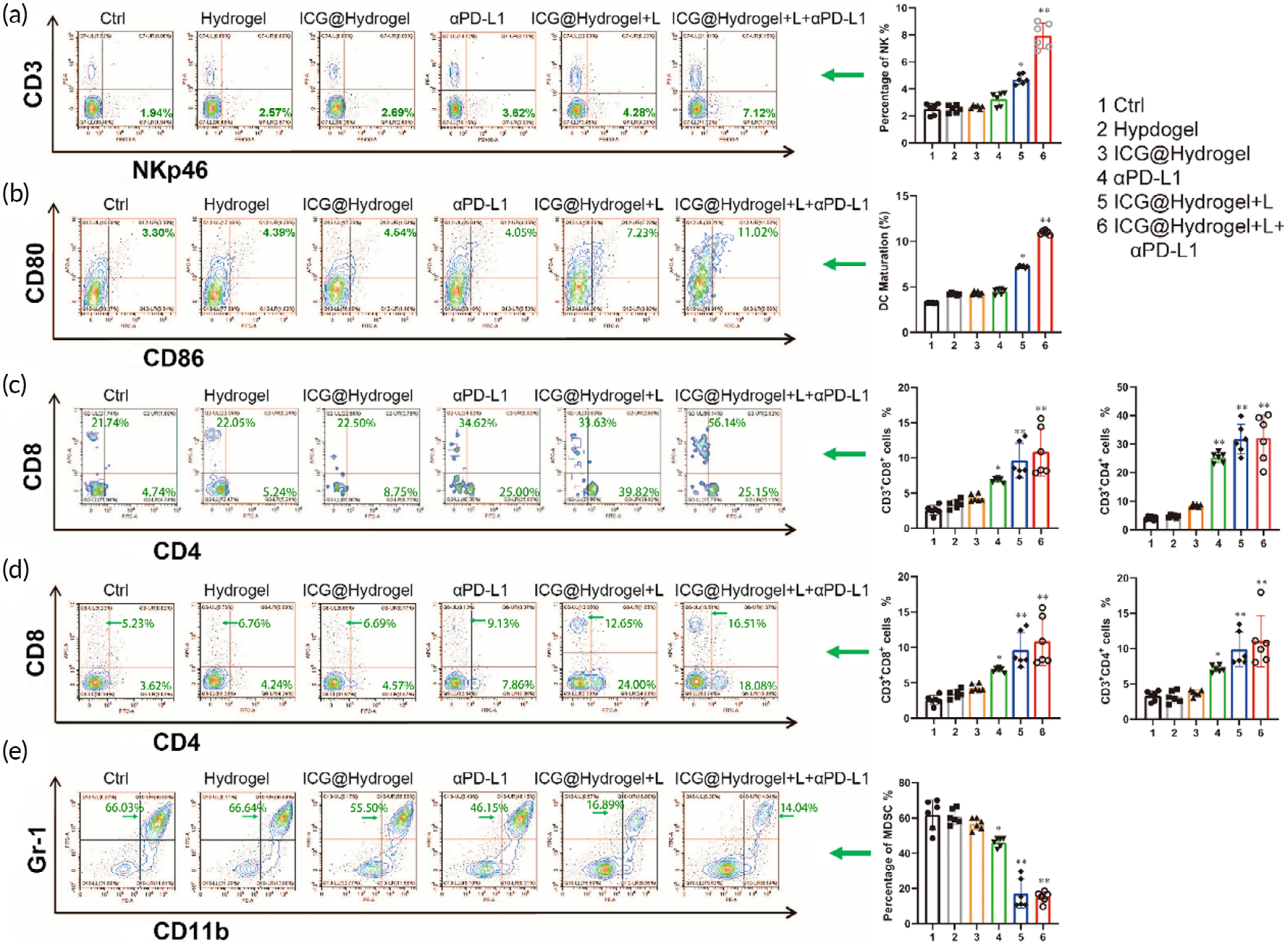
Evaluation of immune activation effect after various treatments. (a) Flow cytometry analysis of NK cells (NKp46^+^CD3^−^) in spleens after various treatments. Quantification of the NK cells in spleens. (b) Flow cytometry analysis of activated DCs (CD11c^+^CD80^+^CD86^+^) in lymphocyte after various treatments. Quantification of mature DCs in lymphocyte. (c) Representative flow cytometric plots of T cells in post‐resection tissue gating on CD3+ cells in each group. Quantification of the CD4^+^ and CD8^+^ T cells in CD3^+^ T cells of tumors. (d) Flow cytometry analysis of CD3^+^CD4^+^ and CD3^+^CD8^+^ T cells in spleens after various treatments. Quantification of the CD4^+^ and CD8^+^ T cells in spleens. (e) Flow cytometry analysis of MDSCs (CD11b^+^Gr‐1^−^) in spleens after various treatments. Quantification of the MDSCs cells in spleens. Data are presented as the mean ± SD (*n* = 6) for (a–e). (Ctrl = no treatment; Hydrogel = granular hydrogel without ICG treatment; ICG@Hydrogel = ICG‐locking gel treatment; αPD‐L1 = αPD‐L1 treatment; ICG@Hydrogel+L = ICG‐locking gel and irradiation treatment; ICG@Hydrogel+L+αPD‐L1 = ICG‐locking gel and irradiation treatment combined with αPD‐L1). (*p < 0.05, **p < 0.05, and ***p < 0.001).

To further elucidate the underlying mechanisms of the antitumor efficacy, we analyzed the immune cell in the main immune organs by flow cytometry. Both innate and adaptive immune responses are indispensable for antitumor immunotherapy.^36^ As shown in Figure 9a, the number of NK cells in spleen was significantly increased in the ICG@Hydrogel+L+αPD‐L1 group, suggesting that innate immunity was activated by PTT. In addition, we further investigated dendritic cells in lymph nodes, which play key roles in activating adaptive antitumor response. Our data indicated that ICG@Hydrogel+L(2.19‐fold) and ICG@Hydrogel+L+αPD‐L1(3.33‐fold) groups effectively promoted DCs mature compared with other groups (Figure 9b). Apart from that, an increased population of CD3^+^CD8^+^T and CD3^+^CD4^+^T cells of tumor and spleen tissues were detected, especially in mice treated with ICG@Hydrogel+L+αPD‐L1 among all the groups, indicating that ICG‐locking gel implantation combined with PD‐L1 triggers T cell significantly mediated adaptive antitumor immune response (Figure 9c,d, Figure S12).

Owing to the immunosuppressive post‐resection microenvironment, the activity of antitumor leukocytes, such as DCs, NK cells, and cytotoxic T lymphocytes, might be suppressed, leading to tumor recurrence and metastasis.^37^ MDSC has the inhibitory function of immune cells, especially T cells. MDSCs could inhibit T cell proliferation and function by down‐regulating the expression of IL‐2, INF‐γ, and granzyme B.^38^ MDSC also reduces the number of CD8^+^ T cells and inhibits its cytotoxicity on tumor cells.^39^, ^40^ As shown in Figure 9e, the number of MDSC notably dropped after treatment with ICG@Hydrogel+L (75%) and ICG@Hydrogel+L+αPD‐L1(80%) groups, which suggested that ICG‐locking gel with αPD‐L1 could reprogram a postsurgery immunosuppressive TME into an immunostimulatory one.

These data together confirmed that our designed ICG‐locking gel‐mediated PTT combined with αPD‐L1 produced a practical paradigm for preventing tumor recurrence and diminishing distance metastasis after surgery.

### 3.7 Antilung metastatic tumors and long‐term immune memory effect

**FIGURE 10:**
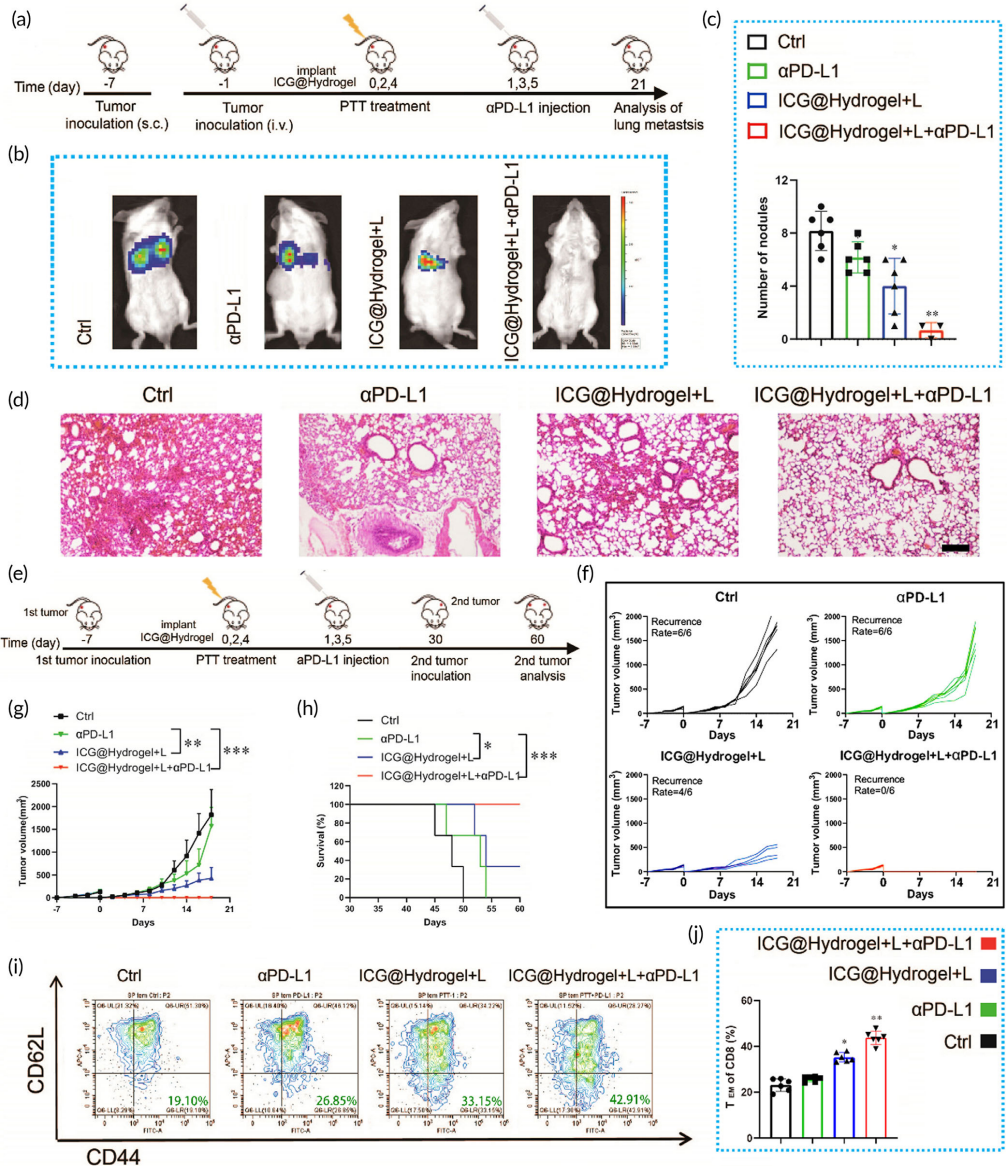
Anti‐lung metastatic tumors and long‐term immune memory effect. (a) Schedule of tumor inoculation and treatment of lung metastasis model. (b) In vivo bioluminescence images to evaluate the growth of lung metastasis after various treatments. (c) Quantification of pulmonary nodules in mice. (d) H&E staining of lung tissue from different groups (bar scale: 200 μm). (e) Schedule of tumor inoculation and treatment of long‐term recurrence model. (f) Individual tumor growth curves of the mice. (g) Average tumor growth kinetics of the mice after various treatments. (h) Survival curves of mice after tumor rechallenge. (i) Flow cytometry analysis of TEM (CD3+CD8+CD44+CD62L‐) in spleens after various treatments. Quantification of the TEM (J) in spleens. Data are presented as the mean ± SD (*n* = 6) for (c, h, j). (Ctrl = no treatment; αPD‐L1 = αPD‐L1 treatment; ICG@Hydrogel+L = ICG‐locking gel and irradiation treatment; ICG@Hydrogel+L+αPD‐L1 = ICG‐locking gel and irradiation treatment combined with αPD‐L1). (*p < 0.05, **p < 0.05, and ***p < 0.001).

Tumor metastasis as well as recurrence after surgery are the main factors for poor survival rate in CRC. To further verify the antimetastatic tumor activity of ICG‐locking gel that combined with αPD‐L1, the lung metastatic tumor model was established. The primary tumors were inoculated on day −7, and lung metastasis was established by injecting CT26 cells via tail vein on day 7 (Figure 10a). According to the luciferase intensity of mice, the ICG@hydrogel+L+αPD‐L1 group showed less lung metastatic tumor lesions as compared with that in other groups, which was further confirmed by H&E staining of lung tissues (Figure 10b–d).

To examine the long‐term immune memory effects, we established recurrent CT26 tumor models. To the recurrence of the tumor after the indicated treatment, the tumors were incompletely eradicated by ICG‐locking gel and irradiation treatment or surgery (with a residue of ≈1% of the original volume) after the treatment for 3 weeks (Figure 10e). The mice receiving ICG‐locking gel and irradiation treatment were better protected from local tumor recurrence (with a recurrence rate of 40%) (Figure 10f), and displayed slower growth of the tumor (Figure 10g), compared with untreated and αPD‐L1 groups. Notably, mice in the ICG@hydrogel+L+αPD‐L1 group had no tumor recurrence and exhibited the highest survival rate, as compared with other groups. The survival time of mice in the combination group was also significantly prolonged when compared to other groups (Figure 10h). The effector memory T cells (CD3+CD8+CD44+CD62L−) (Tem) in the spleens of the mice were examined by flow cytometry. ICG‐locking gel and irradiation and αPD‐L1 treatment generated much more Tem cells than the other groups (Figure 10i, j, Figure S13), suggesting that the combination treatment evoked an immune memory response.

## 4 DISCUSSION

Since surgical resection cannot completely eradicate the tumor tissue, which inevitably increases the risk of local recurrence and metastasis, thus poses a challenge to the clinical treatment. Recently, hydrogel‐based immunotherapy has become a promising option for postoperative tumor therapy treatment, because of its good biocompatibility, controllability, injectability, high drug loading, low invasiveness, prolonged drug release, and multifunctionality.^41^, ^42^ Especially, hydrogels loaded with photothermal functional components could focus PTT on the surgical site, when in combination with immunotherapy not only kills local tumor cells, but also stimulate systemic antitumor immune response, thus inhibits the recurrence/metastasis and avoids system side effects. However, hydrogels used for postoperative tumor therapy treatment need to meet high‐performance requirements, including injectability for applying to complex and irregular organizational environments, strong tissue adhesion in the humoral environment of most organs for fixing, photothermal functional components trapping for sustained PTT, as well as suitable degradation rate and good biocompatibility.

Although photothermal therapy combined with checkpoint inhibitor has been reported to show outstanding curative effect, various photothermal materials have not been verified in the clinic. Thus, the present chose ICG as the photothermal component, which has been approved by U.S. Food and Drug Administration (FDA) for clinical application, possessing outstanding photothermal conversion efficiency upon NIR irradiation, as well as definite biocompatibility and biosafety.^17^ However, ICG alone cannot be fixed on the surgical site for its water‐soluble feature and rapid scavenging in vivo. In order to ensure that ICG can play an adequate role in photothermal therapy after surgery, this study designed multi‐levels of guarantees. For one level, a tissue adhesive function was designed into hydrogel to ensure the fixing of the ICG hydrogel. Dialdehyde starch could provide abundant hydrogen bonding and Schiff Base bonding with tissue to realize tissue attachment. In addition, thermosensitive P(SBMA‐*co*‐NIPAM) at 37°C showed hydrophobic characteristics to prevent excessive water molecules from destroying the adhesion between gel and tissue. The P(SBMA‐*co*‐NIPAM) gelled to further enhance the granular hydrogel itself, so as to improve the adhesive force and hydrogel stability. For the other level, hydrogel adhered on the surgical site should prevent water‐soluble ICG from releasing. This was attributed to the presence of P(SBMA‐*co*‐NIPAM), which could form electrostatic attractive interactions and hydrophobic interactions with ICG at 37°C, significantly improved the retention of ICG in granular hydrogel and limited the diffusion of ICG. Besides, the degradation was as long as 5 weeks, revealing that the ICG granular gel was stable enough for the PTT course. Such multiple guarantees made the ICG stable and firmly fixed at the surgical site to play the role of PTT.

Besides, the PTT guarantee, convenient application onto surgical site was also one of the characteristics of the present ICG‐locking granular gel. Previous studies including ours showed that granular hydrogel, a kind of bulk hydrogel formed by densely assembled microparticles, can exhibit properties of thixotropy under shear force, thus performing injectability through the design of particle interaction. The flowing granular gel can recover back to solid gel immediately after injection.^43^, ^44^ Thus, the granular hydrogel is a “solid‐to‐solid” injectable system. Its thixotropy makes it act like the commonly used creams in dermatology that can be easily applied to the whole tissue area via spread without further extra external stimuli for gelation.^45^

Combined photothermal and immunotherapy demonstrates great potential in the treatment of cancer postsurgical relapse and metastasis. Our strategy combined photothermal tumor ablation via ICG‐locking hydrogel together with anti‐PD‐L1 checkpoint blockade therapy to achieve superior antitumor efficacy in CRC. Generally speaking, the combination therapy with ideal inhibition effect on the growth of primary tumor, and triggered an abscopal effect controlling distant tumor growth, as well as the immune memory protection to prevent tumor relapse may be explained by three mechanisms. First, PTT of ICG‐locking gel kills local tumor cells by inducing apoptosis and necrosis, which could also generate tumor‐associated antigens to elicit specific immune responses. Second, our gel‐mediated PTT could effectively induce ICD in CRC cells by stimulating CRT exposure and releasing HMGB1 and ATP, as well as promote in vitro DCs maturation, thus promoting antitumor cell immunity. Third, our strategy could reprogram the postsurgery immunosuppressive TME by promoting DCs maturation, increasing the percentage of NK cells, enhancing the infiltration of effector T cells, and decreasing the number of MDSC in mice's main immune organs, leading to maximized antitumour immunity of anti‐PD‐L1 for synergistic cancer immunotherapy.

Distinguished from previously reported hydrogel‐based immunotherapy strategies, our hydrogel platform locked FDA‐approved ICG with definite photothermal function and biosafety for PTT combined with anti‐PD‐L1, which is more suitable for clinical translation and application. The limitations of this hydrogel‐based photothermal therapy for CRC are probably as follows: (1) it is hard to keep the hydrogel immobilized at the surgical site for a long time result from the unique position of the colon, which is the fundamental challenge for most hydrogel‐based photothermal therapy in CRC; (2) the hydrogel is likely to interfere with postoperative healing; (3) because of the poor tissue penetration of NIR light in vivo, it is possibly difficult for PTT to achieve great antitumor effect. Therefore, it is urgent to establish hydrogels that not only have great biocompatibility but also could adhere tightly to colon tissue. It is worth mentioning that, endoscope‐based clinical devices with imbedded laser optical fibers could be used to treat tumors located deeply inside the body to overcome the limitation of the penetration depth of light in tissues with NIR lasers.

## 5 CONCLUSION

In summary, we demonstrated a combined photothermal immunotherapy strategy by spreading ICG‐locking gel to tumor resection cavity for preventing CRC postsurgical relapse and metastasis. With the presence of P(SBMA‐*co*‐NIPAM), ICG was highly retained in hydrogel. With the photothermal action of ICG‐locking gel on the postsurgical site, the combination treatment not only elicited systemic antitumor immunity but also reprogramed an impressive microenvironment, preventing local tumor growth, distance metastasis, and long‐term recurrence. Considering the biosafety, biodegradability, and well‐performed tissue adhesive ability, the ICG‐locking gel is not only suitable for postoperative treatment of CRC but also for other human cancers that can be resected.

## AUTHOR CONTRIBUTIONS

**Yuan Zeting:** Data curation (equal); investigation (equal); methodology (equal); project administration (equal); supervision (equal); validation (equal); writing – original draft (equal); funding acquisition (equal). **Ma Shuli:** Investigation (equal); methodology (equal); validation (equal); writing – original draft (equal). **Li Yue:** Methodology (equal); validation (equal); writing – review and editing (equal). **Fang Haowei:** Methodology (equal); resources (equal). **Shang Jing:** Methodology (equal); validation (equal). **Zhan Yueping:** Formal analysis (equal); software (equal). **Wang Jie:** Formal analysis (equal); resources (equal). **Chen Teng:** Conceptualization (equal); resources (equal). **Deng Wanli:** Conceptualization (equal); funding acquisition (equal); resources (equal); validation (equal). **Kunxi Zhang:** Conceptualization (equal); funding acquisition (equal); project administration (equal); resources (equal); writing – review and editing (equal). **Yin Peihao:** Conceptualization (equal); funding acquisition (equal); project administration (equal); resources (equal); writing – review and editing (equal).

## CONFLICT OF INTEREST STATEMENT

The authors declare no conflict of interest.

### PEER REVIEW

The peer review history for this article is available at https://www.webofscience.com/api/gateway/wos/peer‐review/10.1002/btm2.10576.

## Supporting information


**Data S1.** Supplementary Information.

## Data Availability

The raw/processed data required to reproduce these findings cannot be shared at this time as the data also forms part of an ongoing study.
