# Supplementary material for: Tissue adhesive indocyanine green‐locking granular gel‐mediated photothermal therapy combined with checkpoint inhibitor for preventing postsurgical recurrence and metastasis of colorectal cancer
Source: Bioeng Transl Med. 2023 Sep 18;8(6):e10576. doi: 10.1002/btm2.10576 (PMC10658503; doi:10.1002/btm2.10576)

**Supporting Information**

**Tissue adhesive indocyanine green-locking granular gel mediated photothermal therapy combines with checkpoint inhibitor for preventing post-surgical recurrence and metastasis of colorectal cancer**

*Zeting Yuan* *^a,b,f,g,1^, Shuli Ma ^a,b,f,1^, Yue Li ^a,b,1^, Haowei Fang ^e^, Jing Shang ^a,b,g^, Yueping Zhan ^a,b,^, Jie Wang ^c^, Teng Chen ^c^, Wanli Deng ^d,^*, Kunxi Zhang ^a,e,^*, Peihao Yin ^a,c,f,^**

*^a^ Interventional Cancer Institute of Chinese Integrative Medicine & Putuo Hospital, Shanghai University of Traditional Chinese Medicine, Shanghai 200060, P. R. China.*

*^b^ Central Laboratory, Putuo Hospital, Shanghai University of Traditional Chinese Medicine, Shanghai 200060, P. R. China.*

*^c^ Department of General Surgery, Putuo Hospital, Shanghai University of Traditional Chinese Medicine, Shanghai 200060, P. R. China.*

*^d^ Department of Oncology, Putuo Hospital, Shanghai University of Traditional Chinese Medicine, Shanghai 200060, P. R. China.*

*^e^ Department of Polymer Materials, School of Materials Science and Engineering, Shanghai University, Shanghai 200444, P. R. China.*

*^f^ Department of Pharmaceutics, School of Pharmacy, East China University of Science and Technology, Shanghai 200237, China.*

*^g^ Shanghai Putuo Central School of Clinical Medicine, Anhui Medical University, Hefei 230032, P. R. China.*

* Corresponding author. W. Deng, E-mail: dwl0707@163.com

* Corresponding author. K. Zhang, E-mail: [zhangkunxi@shu.edu.cn](mailto:zhangkunxi@shu.edu.cn)

* Corresponding author. P. Yin, E-mail: [yinpeihao@shutcm.edu.cn](mailto:yinpeihao@shutcm.edu.cn)

^1^ These authors contributed equally to this work.

**Figure S1:**

Diameter of gelatin microspheres with/without co-polymer.

**
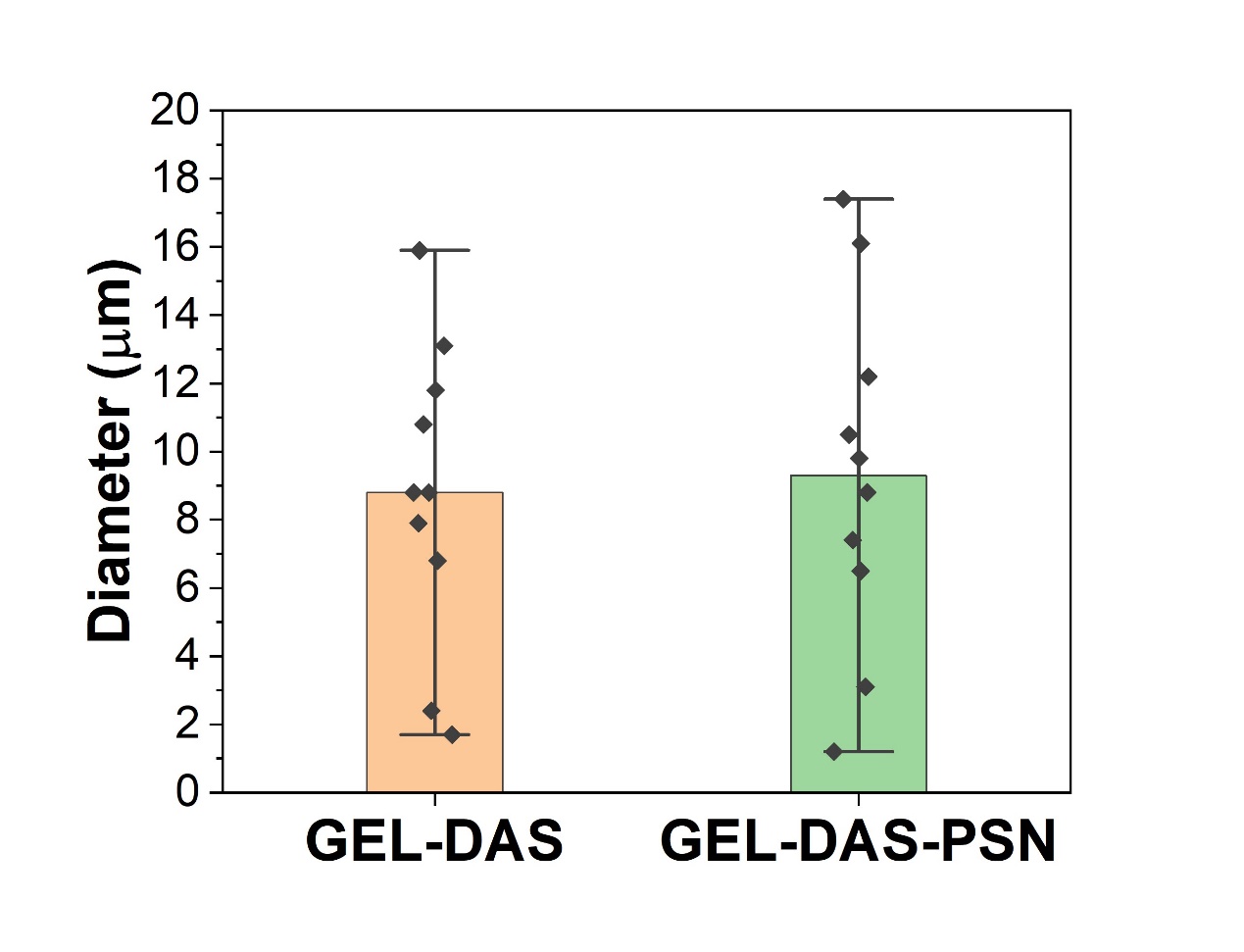
**

**Figure S2:**

Adhesive strength of gelatin microspheres without the starch and gelatin microspheres without the P(SBMA-co-NIPAM) copolymer.

**
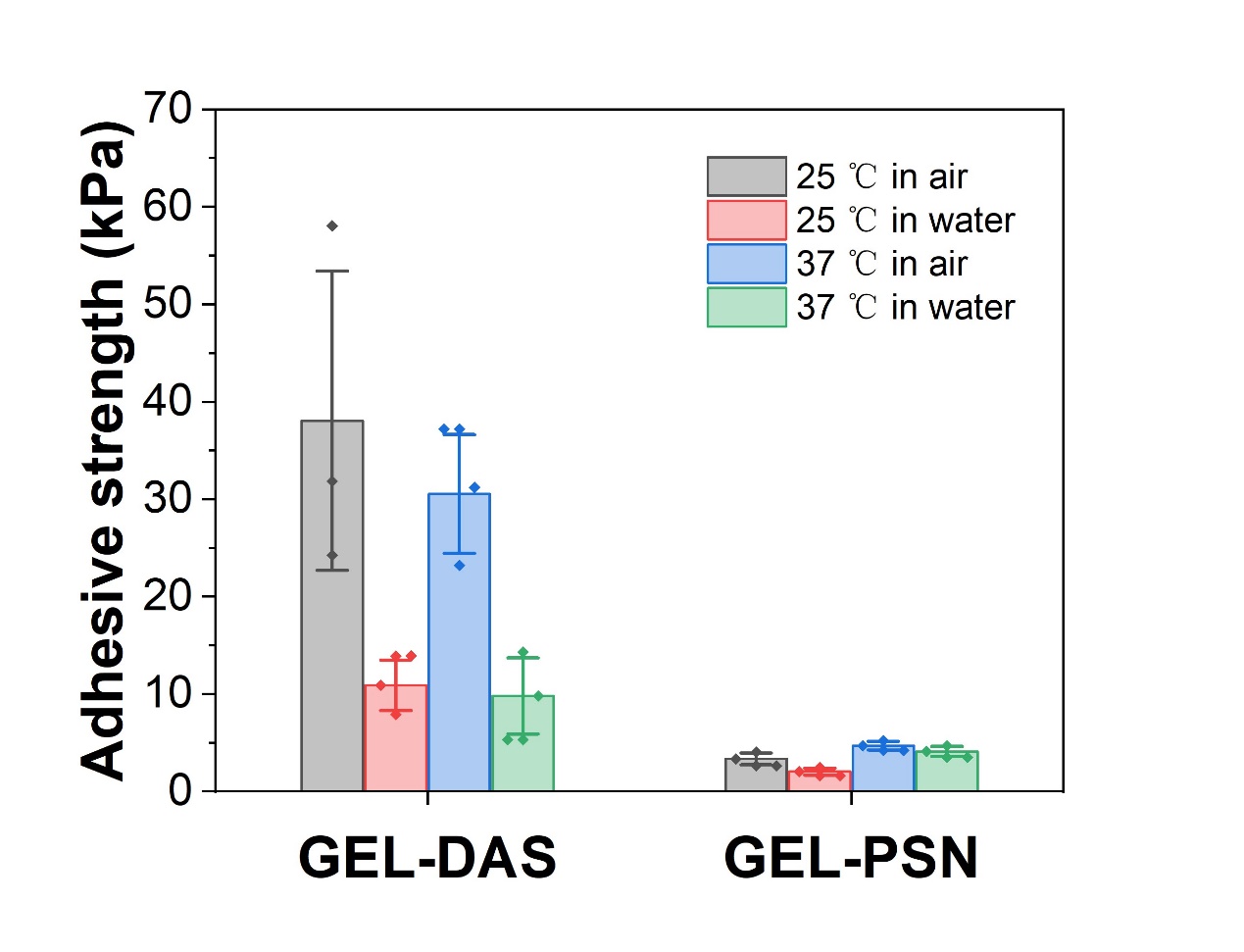
**

**Figure S3:**

*In vivo* ICG release between the ICG@GEL-DAS and ICG@GEL-DAS-PSN groups.

**
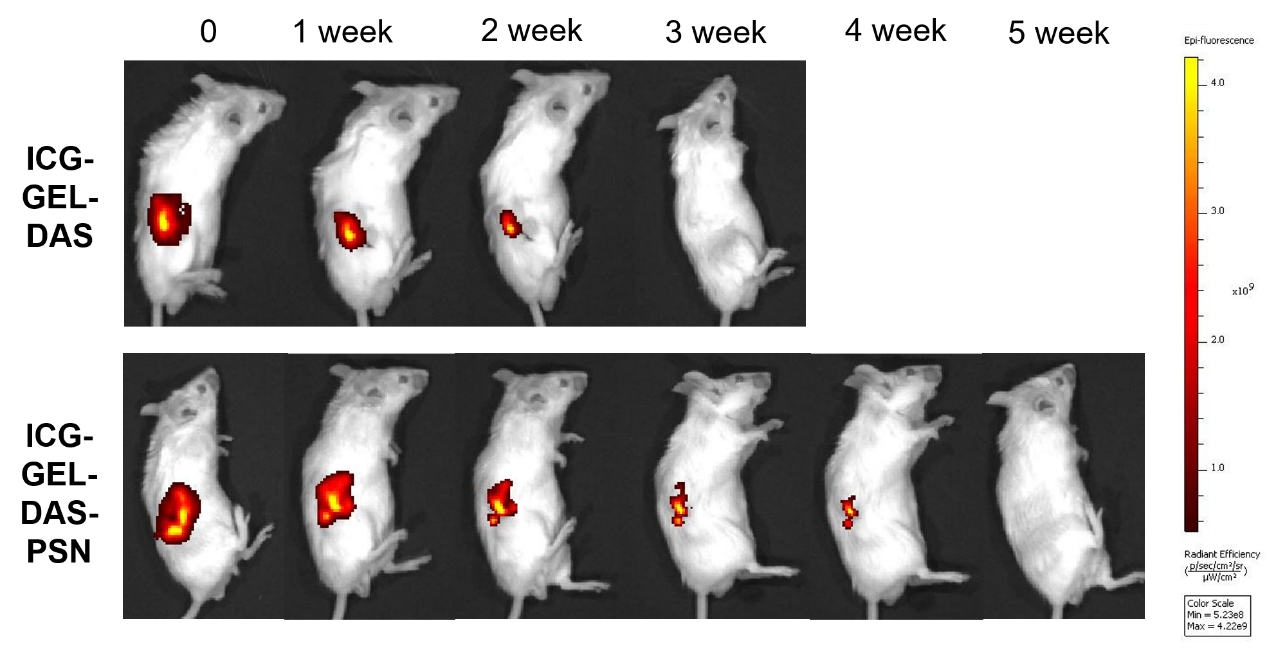
**

**Figure S4:**

Cell viability of HCT116 cells after treatment with ICG solution or ICG-locking gel for 24 h determined by CCK-8 assay.


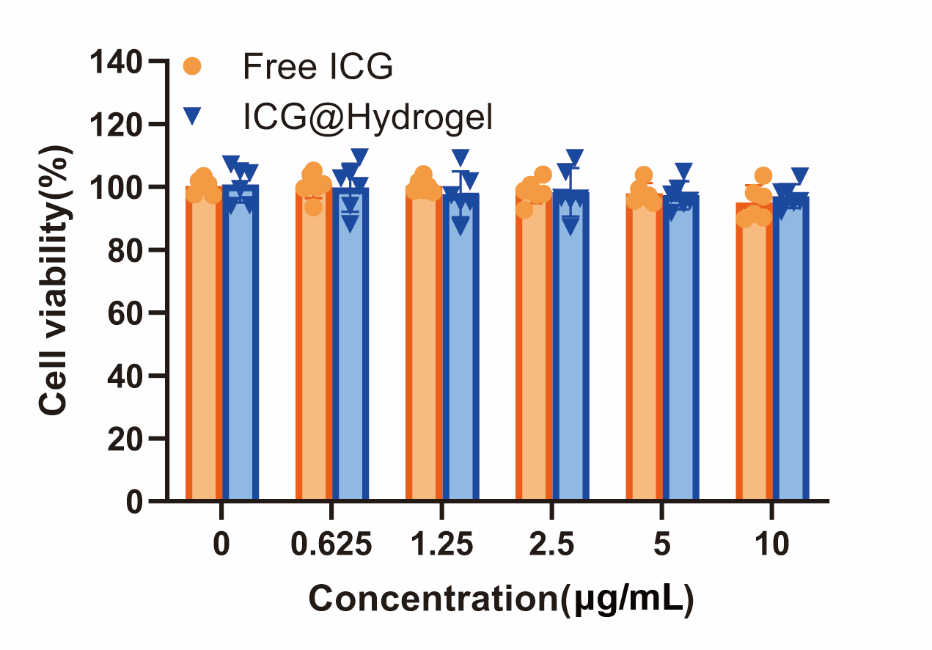


**Figure S5:**

Cell viability of HCT116 cells treated by ICG solution or ICG-locking gel with NIR irradiation for 5 min (808 nm, 1.5 W/cm^2^).


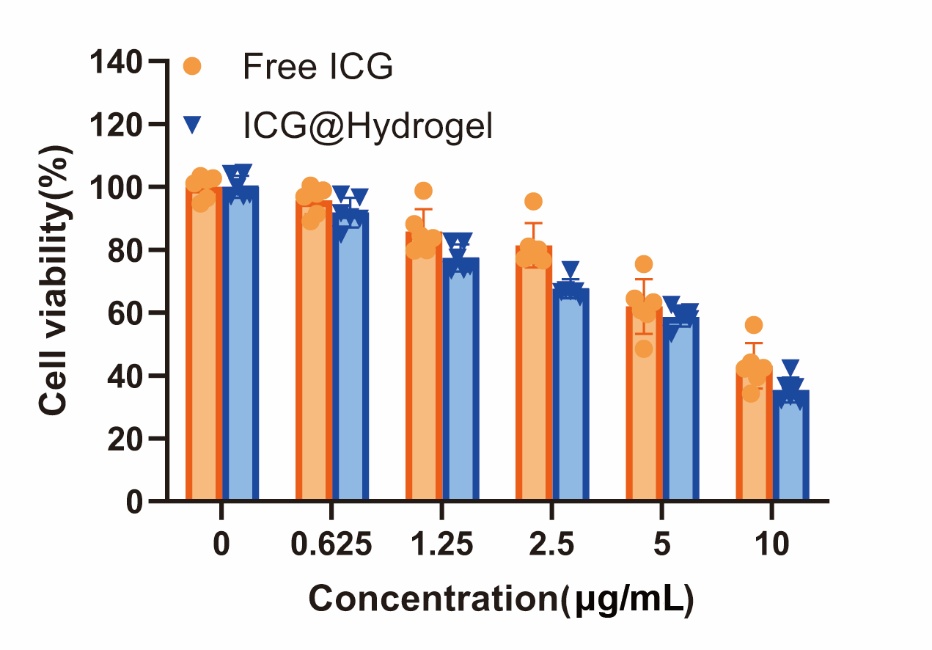


**Figure S6:**

Calcein-AM/PI double staining of HCT116 cells treated with different concentrations of ICG-locking gel with laser irradiation. Scale bars represent 200 µm.


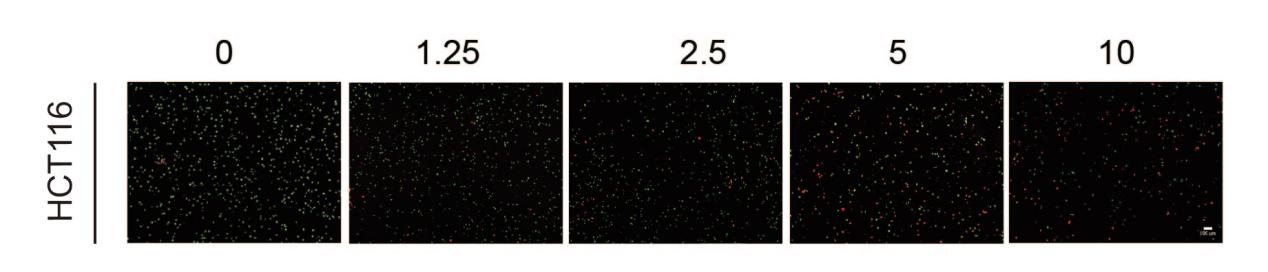


**Figure S7:**

A) Apoptosis analysis of HCT116 cells resulting from combined treatment with different concentrations of ICG-locking gel and NIR irradiation (808 nm, 1.5 W/cm^2^) for 24 h. (B) Quantification for the apoptosis rate of HCT116 cells. Data are presented as the mean ± SD (n = 3). (*P < 0.05, **P < 0.05, and ***P < 0.001).


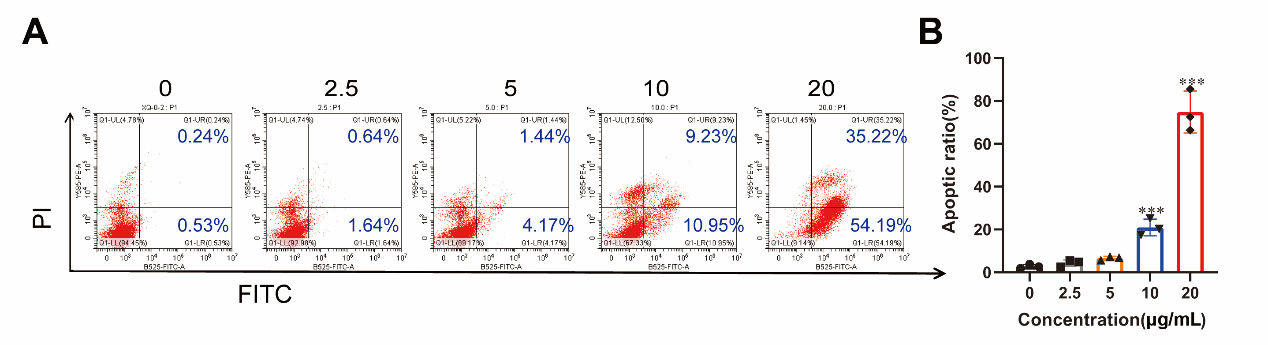


**Figure S8:**

CLSM images (scale bar: 25 μm) of CRT exposure on the surface of HCT116 cells after different treatments with ICG-locking gel and NIR irradiation (808 nm, 1.5 W/cm^2^) for 24 h.


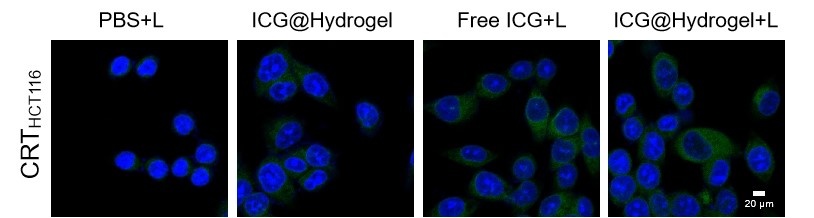


**Figure S9:**

A) Extracellular HMGB1 and intracellular ATP (B) levels of HCT116 cells after different treatments as indicated. Data are presented as the mean ± SD (n = 3). (*P < 0.05, **P < 0.05, and ***P < 0.001).


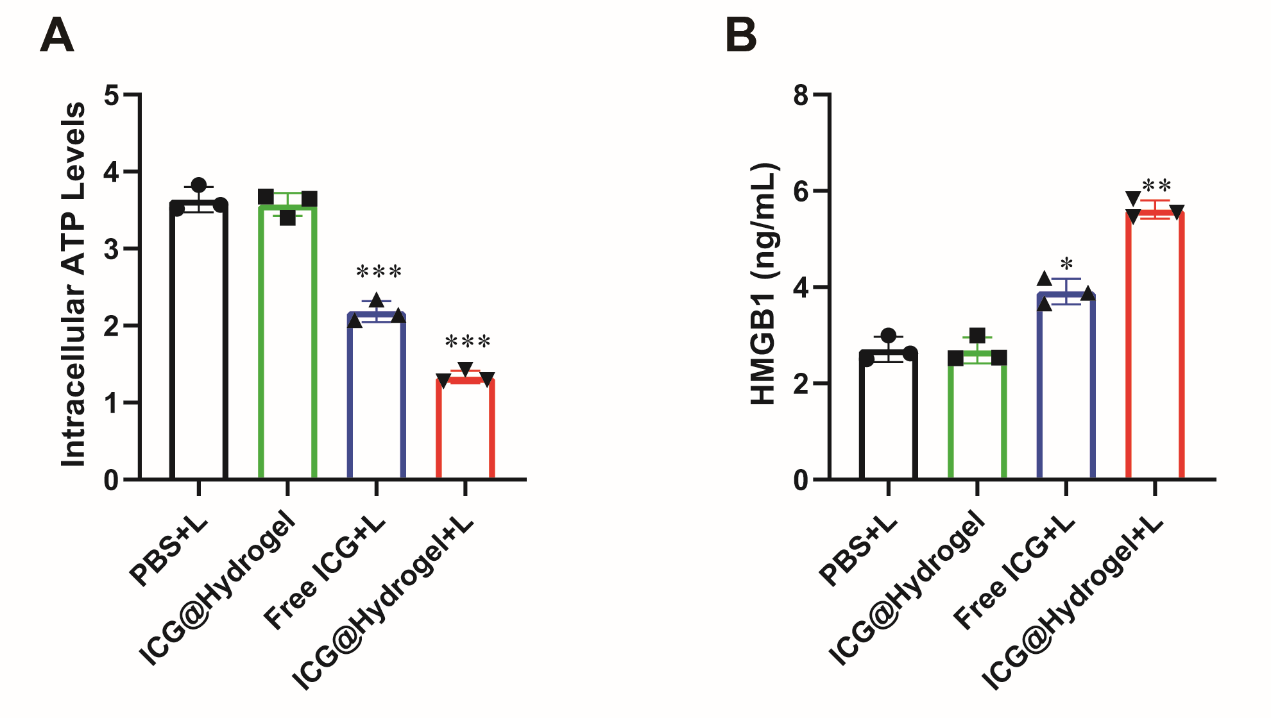


**Figure S10:**

In vitro transwell system experiment. CT26 tumor cells were placed in the upper chamber, and DCs were cultured in the lower chamber. Quantification of CD86 and CD80 expression by flow cytometry for various samples in the in vitro transwell system experiment. Data are presented as the mean ± SD (n = 3). (*P < 0.05, **P < 0.05, and ***P < 0.001).

**
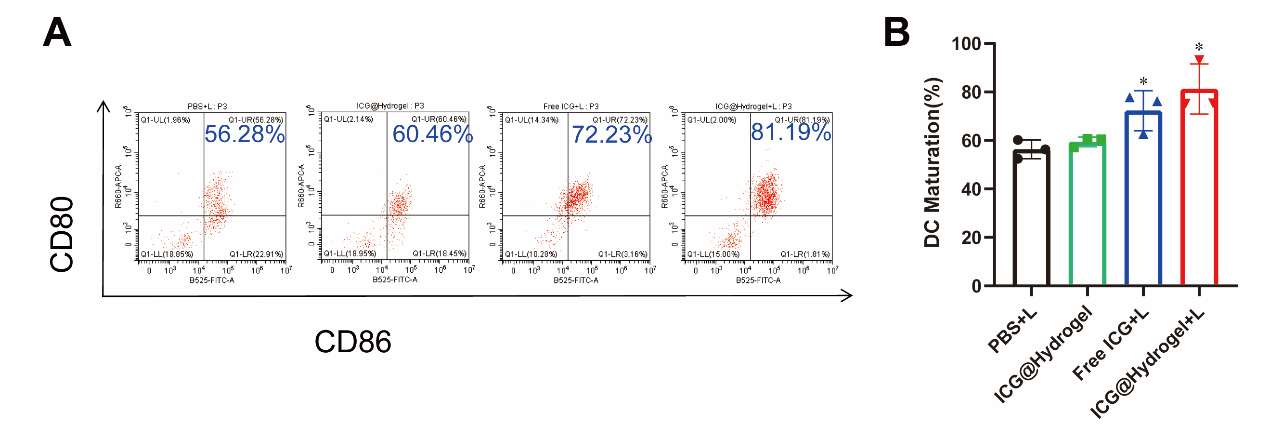
**

**Figure S11:**

Systemic toxicity evaluation. A) Tumor growth curves after the indicated treatments (n=6). B) H&E staining of major organs from different groups (bar scale: 200 μm). (Ctrl=no treatment; Hydrogel=granular hydrogel without ICG treatment; ICG@Hydrogel= ICG-locking gel treatment; αPD-L1=αPD-L1 treatment; ICG@Hydrogel+L=ICG-locking gel and irradiation treatment; ICG@Hydrogel+L+αPD-L1=ICG-locking gel and irradiation treatment combined with αPD-L1)

**
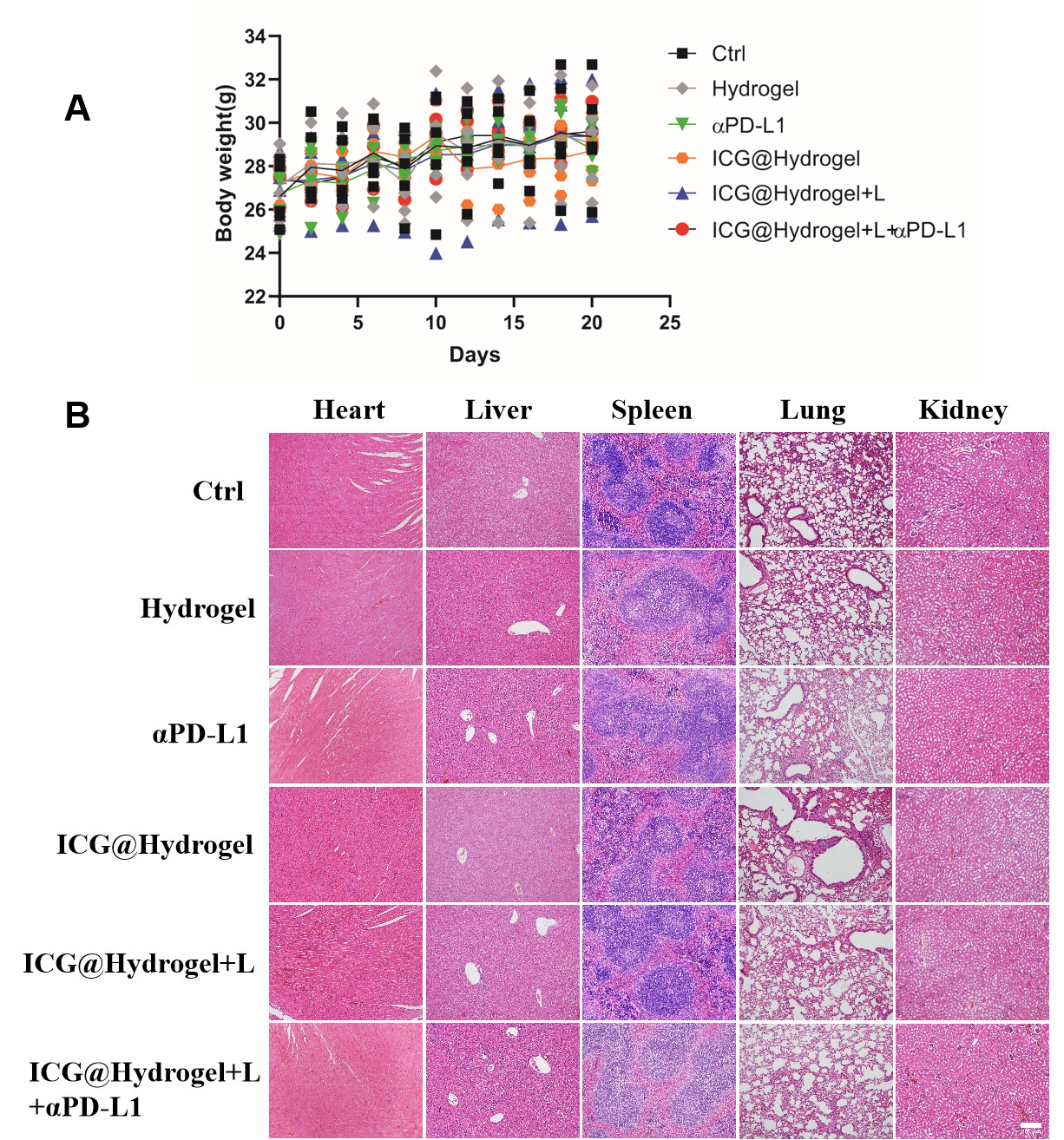
**

**Figure S12:**

1. IFNγ levels, TNFα levels (B) and IL-6 levels (C) in sera from mice isolated 28 days after various treatments. Data are presented as the mean ± SD (n = 3). (*P < 0.05, **P < 0.05, and ***P < 0.001).


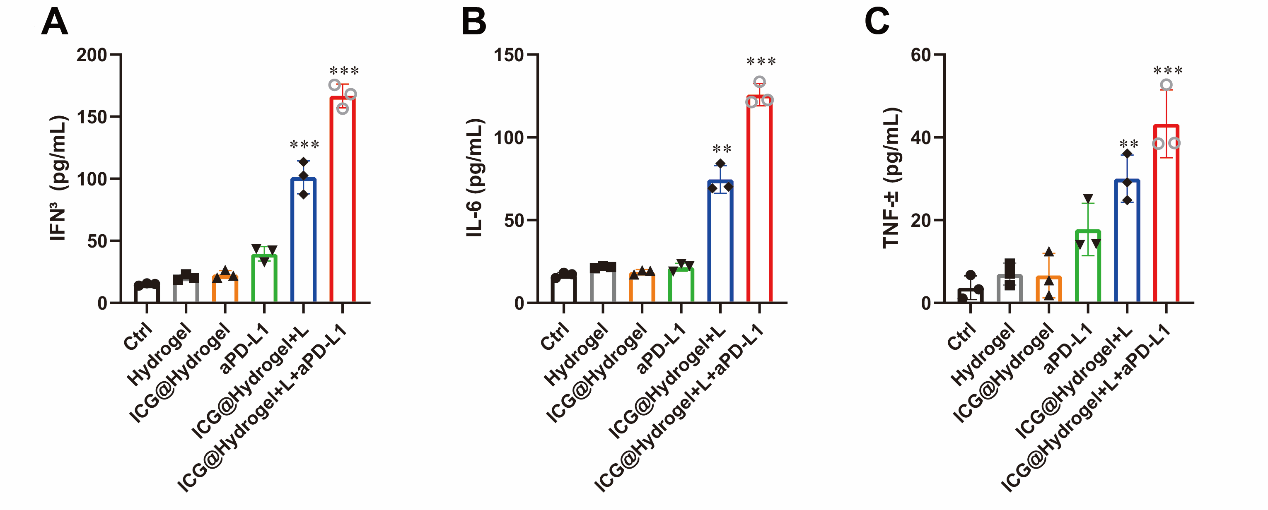


**Figure S13:**

Gating strategy for (A) DCs in vitro, (B) DCs in lymph node, (C) CD8^+^ and CD4^+^T cells in spleen, (D) NK cells in spleen, (E) CD8^+^ and CD4^+^T cells in tumour, （F）Tem in spleen，(G) MDSC in spleen by flow cytometry .


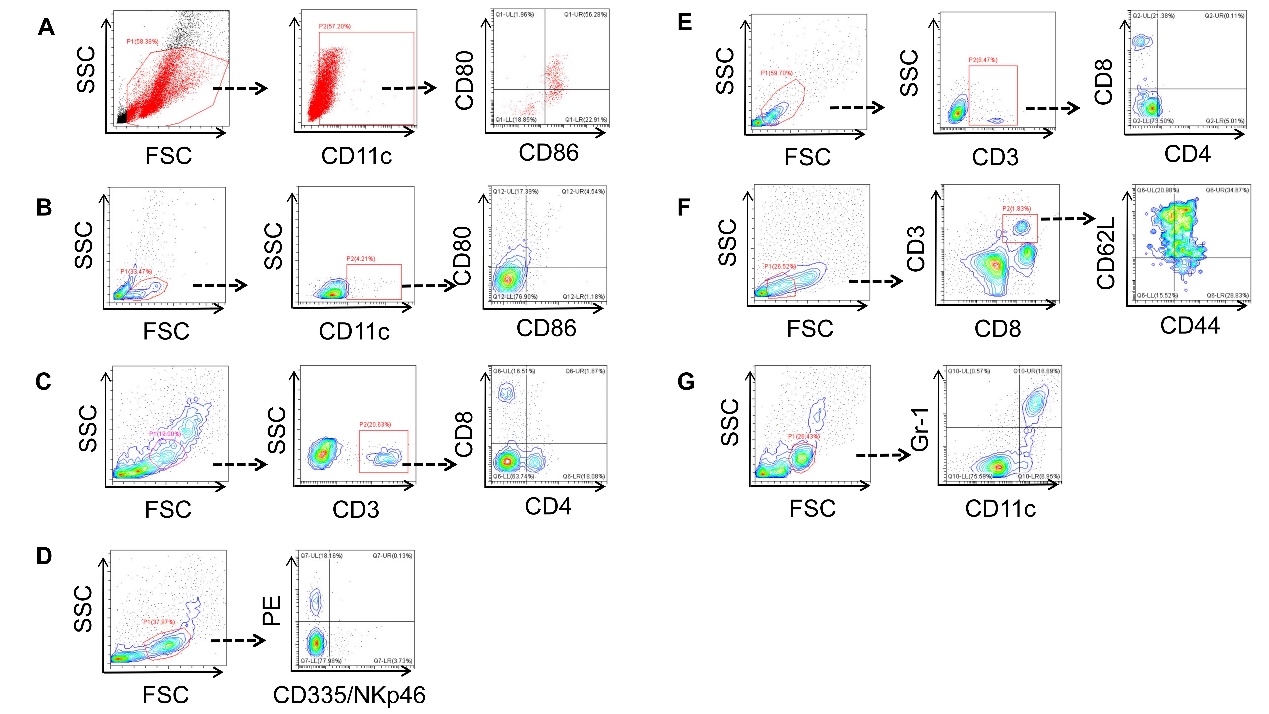

Supplement: Supplementary file 1 — Data S1. Supplementary Information. [file BTM2-8-e10576-s001.docx]
